# Supplementary figures and images for: Standard automated perimetry using size III and size V stimuli in advanced stage glaucoma: an observational cross-sectional comparative study
Source: BMJ Open. 2021 Sep 28;11(9):e046124. doi: 10.1136/bmjopen-2020-046124 (PMC8479951; doi:10.1136/bmjopen-2020-046124)

1A: Standard loss variance

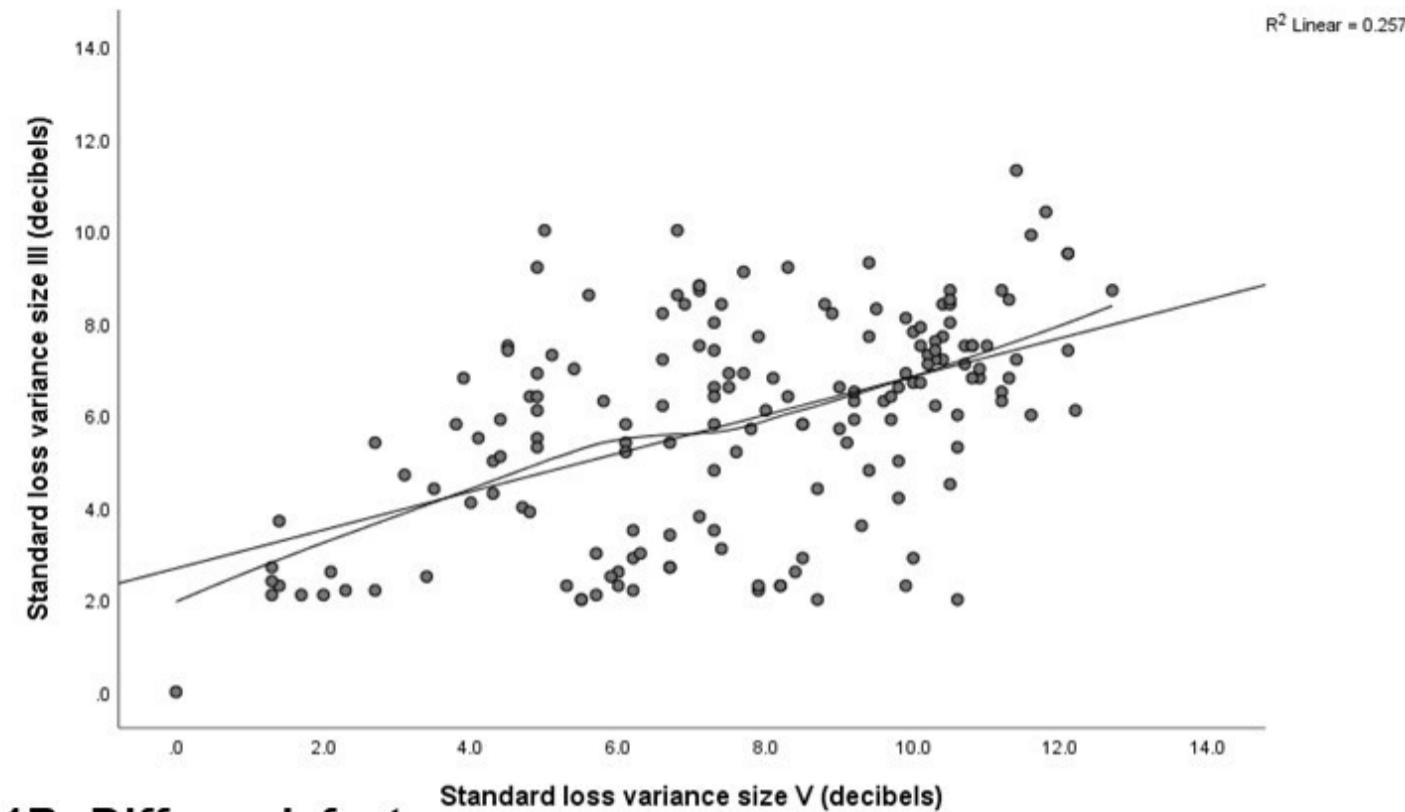

1B: Diffuse defect

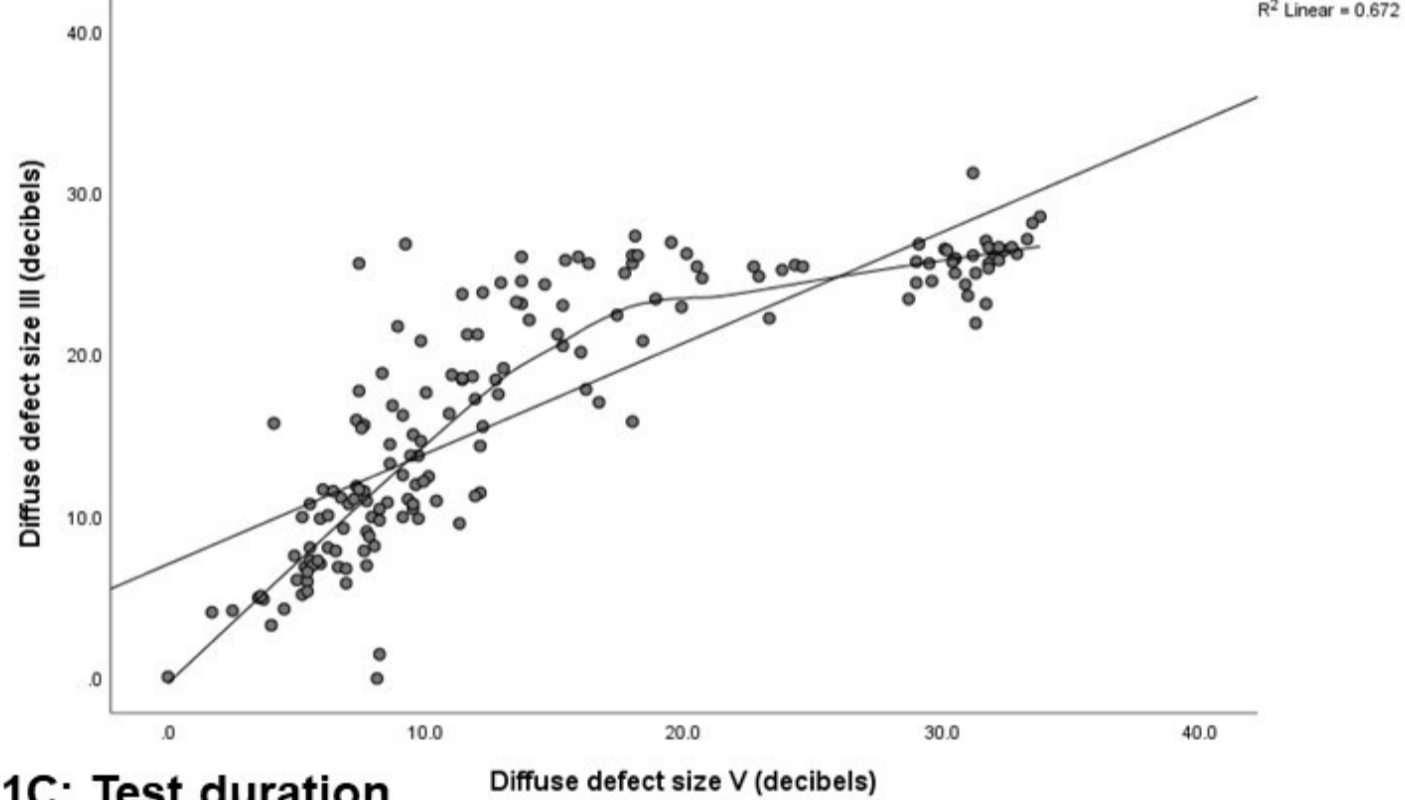

1C: Test duration

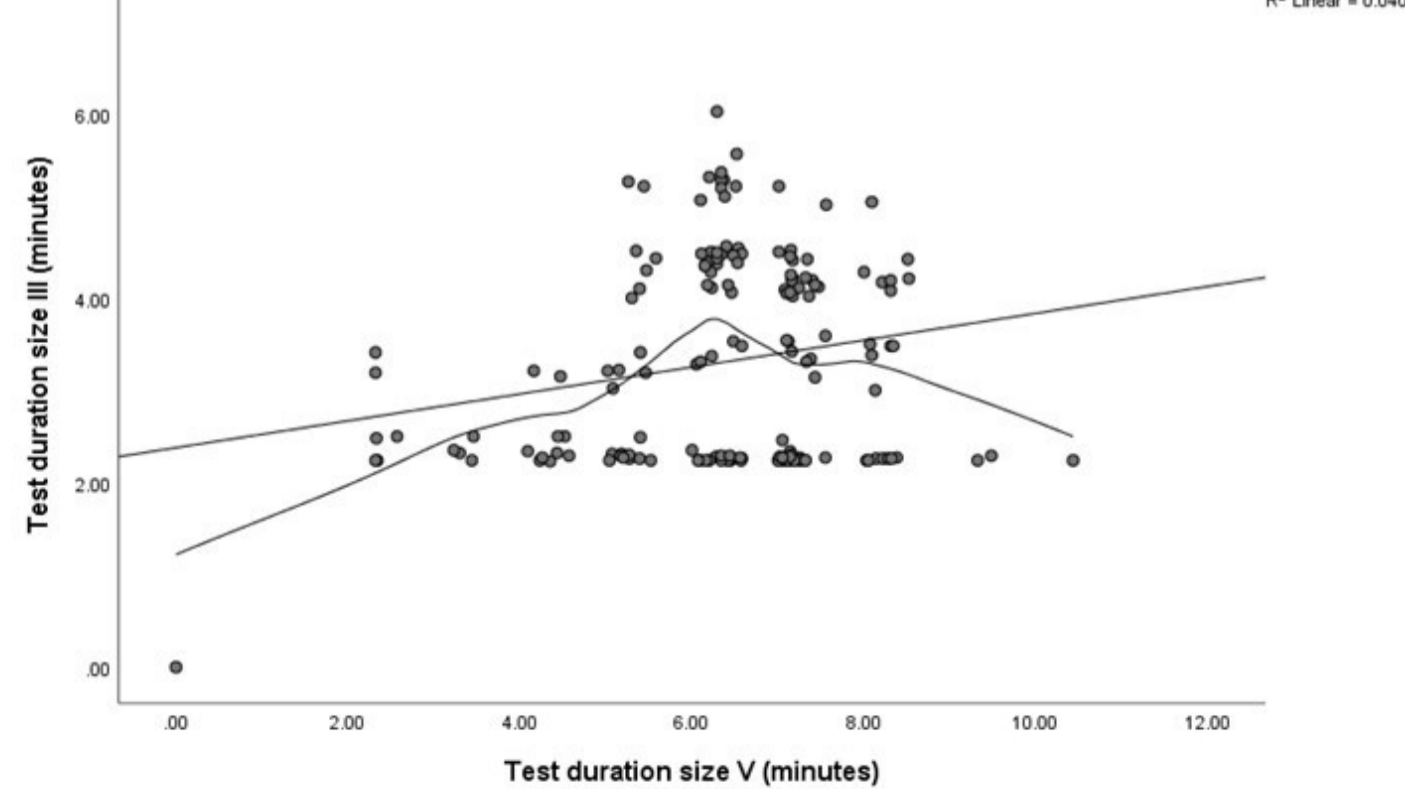

Supplement: Supplementary data [file bmjopen-2020-046124supp001.pdf]

2A: Standard loss variance

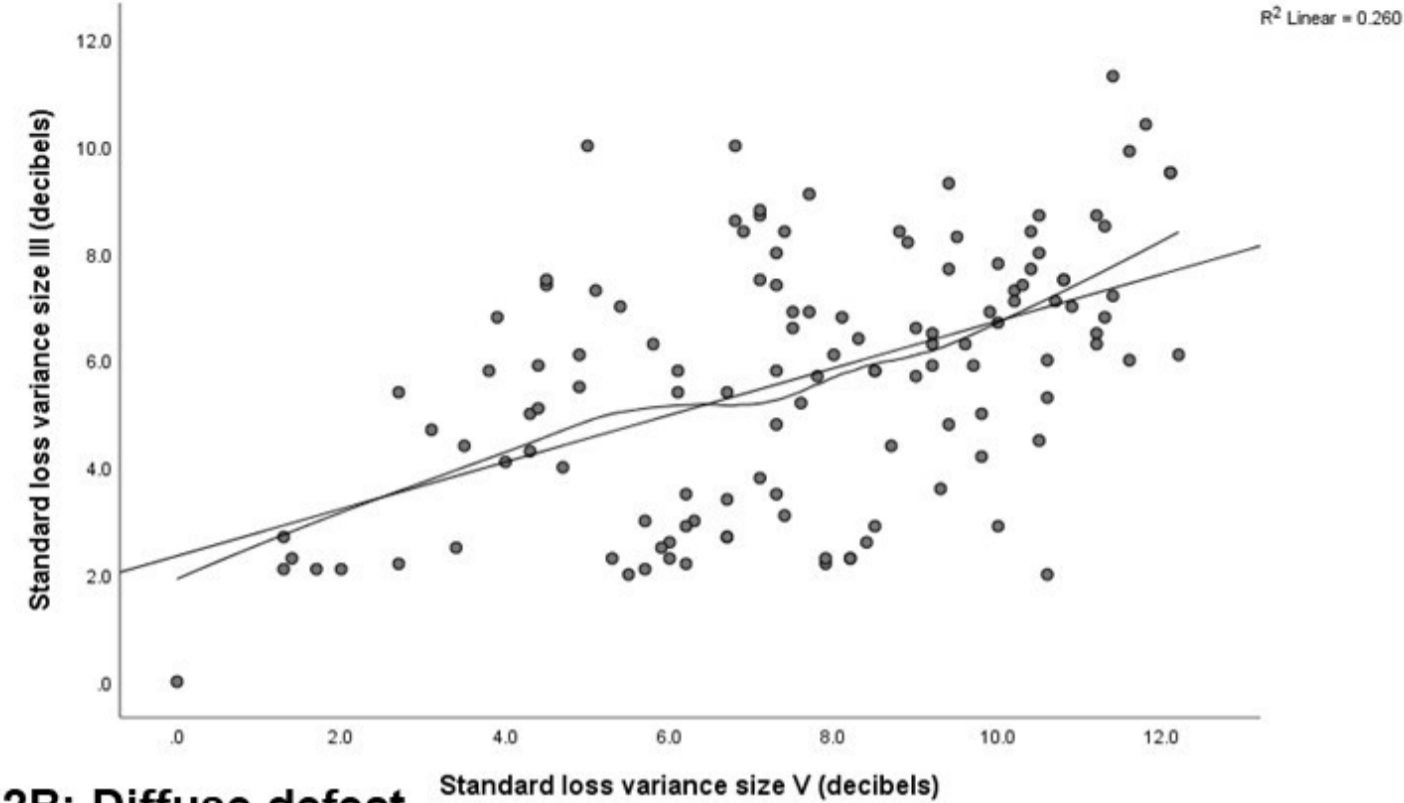

2B: Diffuse defect

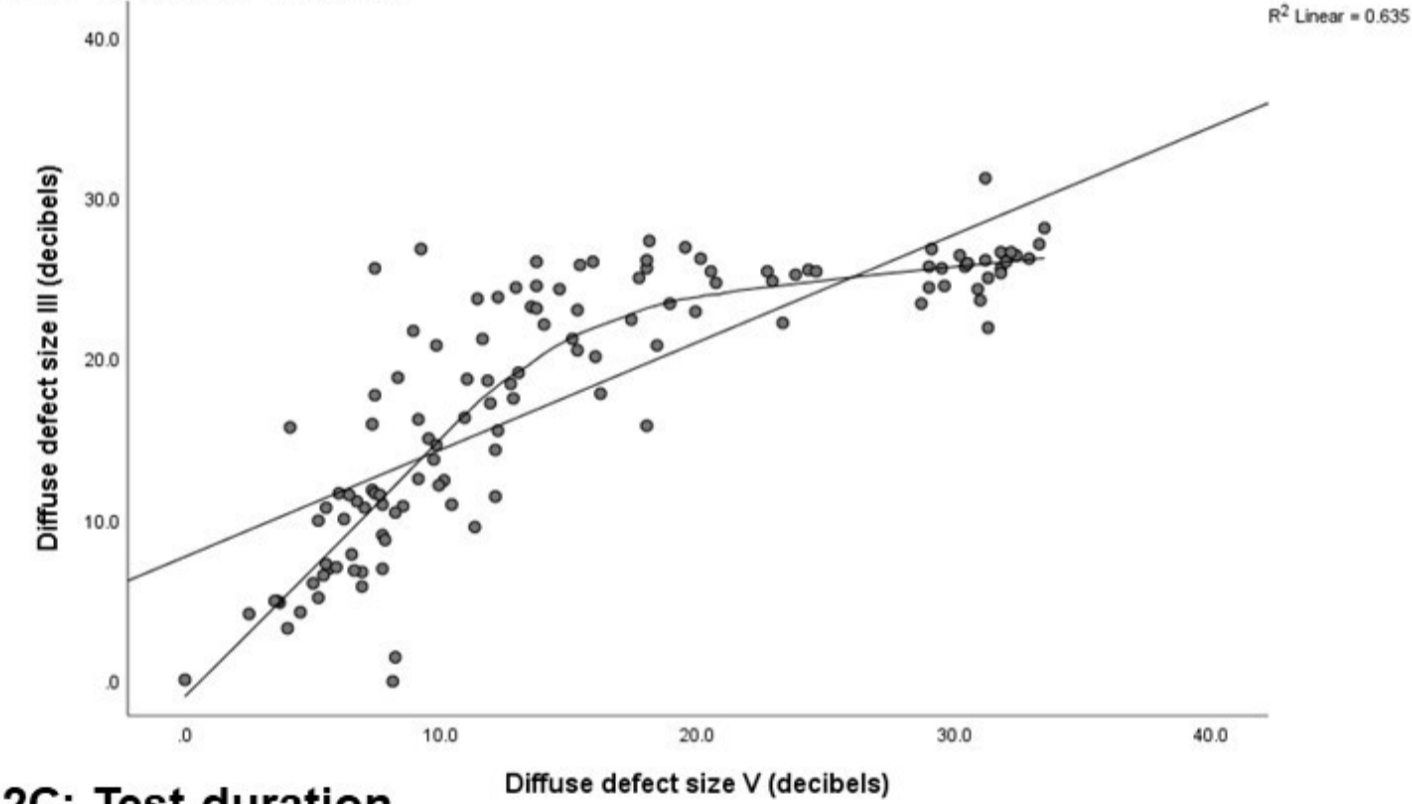

2C: Test duration

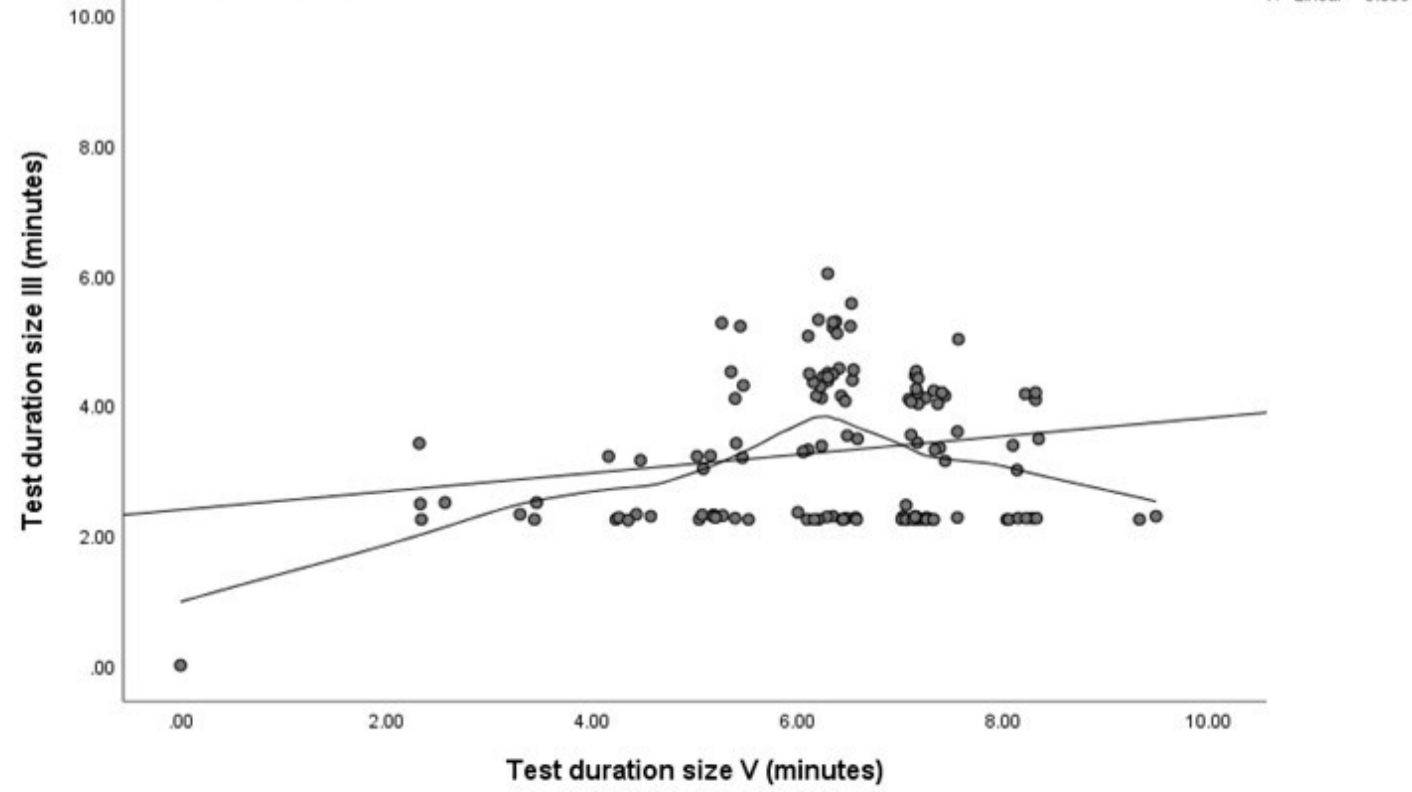

Supplement: Supplementary data [file bmjopen-2020-046124supp002.pdf]
