# Supplementary material for: Standard automated perimetry using size III and size V stimuli in advanced stage glaucoma: an observational cross-sectional comparative study
Source: BMJ Open. 2021 Sep 28;11(9):e046124. doi: 10.1136/bmjopen-2020-046124 (PMC8479951; doi:10.1136/bmjopen-2020-046124)
Supplement: Author's manuscript [file bmjopen-2020-046124.draft_revisions.pdf]

BMJ Open is committed to open peer review. As part of this commitment we make the peer review history of every article we publish publicly available.

When an article is published we post the peer reviewers' comments and the authors' responses online. We also post the versions of the paper that were used during peer review. These are the versions that the peer review comments apply to.

The versions of the paper that follow are the versions that were submitted during the peer review process. They are not the versions of record or the final published versions. They should not be cited or distributed as the published version of this manuscript.

BMJ Open is an open access journal and the full, final, typeset and author-corrected version of record of the manuscript is available on our site with no access controls, subscription charges or pay-per-view fees (http://bmjopen.bmj.com).

If you have any questions on BMJ Open's open peer review process please email info.bmjopen@bmj.com

BMJ Open

Standard automated perimetry comparisons (size III versus size V) in advanced stage glaucoma

Journal:	*BMJ Open*
Manuscript ID	bmjopen-2020-046124
Article Type:	Original research
Date Submitted by the Author:	27-Oct-2020

Complete List of Authors:	Sood, Devindra; Glaucoma Clinic New Delhi, Ophthalmology<br>Czanner, Gabriella; Liverpool John Moores University, Statistics and data science<br>Somerville, Tobi; University of Liverpool Faculty of Health and Life Sciences, Eye and Vision Science<br>Sood, Ishaana; SK Glaucoma Care Foundation, Ophthalmology<br>Rowe, Fiona; University of Liverpool Faculty of Health and Life Sciences, Institute of Population Health Science

Keywords:	Glaucoma < OPHTHALMOLOGY, OPHTHALMOLOGY, Neuro-ophthalmology < OPHTHALMOLOGY

SCHOLARONE™
Manuscripts

I, the Submitting Author has the right to grant and does grant on behalf of all authors of the Work (as defined in the below author licence), an exclusive licence and/or a non-exclusive licence for contributions from authors who are: i) UK Crown employees; ii) where BMJ has agreed a CC-BY licence shall apply, and/or iii) in accordance with the terms applicable for US Federal Government officers or employees acting as part of their official duties; on a worldwide, perpetual, irrevocable, royalty-free basis to BMJ Publishing Group Ltd ("BMJ") its licensees and where the relevant Journal is co-owned by BMJ to the co-owners of the Journal, to publish the Work in this journal and any other BMJ products and to exploit all rights, as set out in our licence.

The Submitting Author accepts and understands that any supply made under these terms is made by BMJ to the Submitting Author unless you are acting as an employee on behalf of your employer or a postgraduate student of an affiliated institution which is paying any applicable article publishing charge ("APC") for Open Access articles. Where the Submitting Author wishes to make the Work available on an Open Access basis (and intends to pay the relevant APC), the terms of reuse of such Open Access shall be governed by a Creative Commons licence – details of these licences and which Creative Commons licence will apply to this Work are set out in our licence referred to above.

Other than as permitted in any relevant BMJ Author's Self Archiving Policies, I confirm this Work has not been accepted for publication elsewhere, is not being considered for publication elsewhere and does not duplicate material already published. I confirm all authors consent to publication of this Work and authorise the granting of this licence.

For peer review only

Standard automated perimetry comparisons (size III versus size V) in advanced stage glaucoma

Devindra Sood MBBS, MS, FACS[1,2,3], Gabriela Czanner PhD CStat[4], Tobi Somerville MBChB, MRes[5], Ishaana Sood, BSc[6], Fiona J Rowe PhD[7]

1. Glaucoma Clinic, New Delhi, India

2. Glaucoma Service, Sadguru Netra Chikatsalya, Chitrakoot, India

3. Head of Academics, Research and Training, Sadguru Netra Chikatsalya, Chitrakoot, India

4. Department of Applied Mathematics, Liverpool John Moore's University, UK

5. Department of Eyes and Vision Science, University of Liverpool, UK

6. SK Glaucoma Care Foundation, New Delhi, India

7. Department of Health Services Research, University of Liverpool, UK

Address for correspondence

Prof Fiona Rowe, Waterhouse Building, Block B, First Floor, University of Liverpool, 1-5 Brownlow Street, Liverpool, L69 3GL

E-mail: rowef@liverpool.ac.uk Twitter: @orthopticbrain

For peer review only

Word Count: 4380

Tables: 2

Figures: 3

Supplementary figures: 2

Short title: Size III vs V perimetry in advanced glaucoma

For peer review only - http://bmjopen.bmj.com/site/about/guidelines.xhtml

Abstract

Objectives: In this study, we sought to evaluate the extent of further visual field that could be assessed when using stimulus size V in standard automated perimetry compared to size III in advanced stage glaucoma and whether cut-off values could be determined for when to switch from size III to size V.

Design: Prospective cross section study.

Setting: Out-patient eye clinic.

Participants: Advanced stage glaucoma defined as stages 3-4.

Intervention: Central static perimetry with Octopus 900 G programme.

Primary and secondary outcome measures: Visual field assessment for right and left eyes with both sizes III and V were undertaken within one clinic visit.

Results: We recruited 126 patients (170 eyes). Mean patient age at assessment was 55.86 years (SD 15.15). Means (SD) for size III versus size V respectively were 6.94dB (5.58) and 12.98dB (7.77) for mean sensitivity, 20.02dB (5.67) and 19.22dB (7.74) for mean deviation, 5.89dB (2.29) and 7.69dB (2.78) for standard loss variance and 3.32 minutes (1.07) and 6.40 minutes (1.43) for test duration. All except mean deviation were significantly different between size III and V tests.

Conclusion: Useful visual field information was obtained with size V stimuli which allowed continued monitoring of these patients that was not possible with size III. Increased test duration, standard loss variance and mean sensitivity were found with size V, as expected, given that more visual responses were obtained with the increased target size. A switch from size III to V may be considered when mean sensitivity reaches 10dB and/or mean deviation reaches 18dB.

Keywords

Standard automated perimetry; Size III; Size V; Glaucoma

Article summary – strengths and limitations of this study

- Size V stimulus in standard automated perimetry has a greater dynamic range than size III stimulus

For peer review only - http://bmjopen.bmj.com/site/about/guidelines.xhtml

- Visual field results of advanced stage 3-4 glaucoma meeting reliability criteria were analysed.
- Sample size calculations were met for recruitment to this study.

Declarations of interest

Funding: This research received no specific grant from any funding agency in the public, commercial or not-for-profit sectors.

Conflicts of interest: FR reports consultancy work for Haag Streit AG and use of perimeters for research from Haag Streit AG and Zeiss Meditec, companies that may be affected by the research reported in the enclosed paper. There are no potential conflicts of interest reported by the other authors.

Data availability: The dataset for this paper is not available for sharing.

Ethics approval: Institution ethical approval (Glaucoma Clinic New Delhi, India) was obtained and patients provided informed consent.

Author contributions: FR and DS conceived this study. DS and IS contributed to data collections. FR and TS contributed to data extraction. FR and GC contributed to data analysis. All authors contributed to writing this paper.

Introduction

Standard automated perimetry using stimulus size III is an internationally accepted visual field assessment for glaucoma [1]. Static threshold perimetry is particularly adept in aiding detection of early visual field loss in glaucoma. For patients with glaucoma, the usual choice of visual field assessment is static perimetry using a threshold strategy to assess the central 24 to 30 degrees of the visual field of right and left eyes individually [1]. In clinical practice a number of issues can arise with these central assessment programmes. As glaucoma progresses and the central visual field deteriorates to the extent that minimal (if any) visual field remains, appropriate and reliable evaluation can become difficult.

For peer review only - http://bmjopen.bmj.com/site/about/guidelines.xhtml

For peer review only

For patients with severe to advanced stage disease, central visual field assessment can show substantial visual field loss and, therefore, is limited in providing useful information about further progression or stability of the visual field loss, or for providing information about the individual's functional visual status. In these patients, further analysis options for visual field assessment must be considered. Where the central visual field island remains, it is possible to target assessment to the central 10 degrees of visual field. Tomairek and colleagues [2] showed a considerable number of eyes with severe glaucoma with absolute visual field defects on 24-2 programme testing showing relative defects involving only some points of the central 10 degrees on the 10-2 programme. Thus they stressed the importance of detailed central testing to underpin intensive treatment to preserve the residual central visual field.

A further alternative is to continue assessment of the central 30 degrees but to increase the stimulus to size V [3-5]. Whilst there is considerable debate over the use of different stimulus sizes in automated perimetry [6-10], from a clinical perspective, size III remains the standard stimulus in mainstream clinical use [1,7]. However, when visual field results using standard automated perimetry show severe loss, use of size V stimulus becomes a consideration.

There are potential issues with size V versus III in early visual field loss as the use of the larger stimulus size could prevent the detection of small localised defects because the large stimulus size stimulates not only the area of visual field loss but also the surrounding, better, areas of visual field [6]. This is not directly relevant to studies that focus specifically on severe visual field loss. With severe visual field loss, when using size III, it is not possible to gather reliable visual field data by increasing stimulus contrast (i.e. lower decibel values) as often there is no response within damaged areas of visual field [6,7]. Thus, a further option is to increase the target size instead.

The useful dynamic range of perimetry is the range of disease severities over which useful measurements can be obtained. With increasing visual field loss, the response within the area of visual field loss becomes more variable because of decreased retinal ganglion cell response rate along with the limited number of stimulus presentations in each location of the test programme [6,7]. Use of size V

For peer review only - http://bmjopen.bmj.com/site/about/guidelines.xhtml

stimulus has been shown to have a greater dynamic range and a similar number of abnormal test locations when compared to the traditionally used size III stimulus [3]. Higher sensitivity is achieved at the same location giving more reliable and less variable estimates of sensitivity at the damaged visual field locations. Gardiner reported that for rapidly progressing eyes, use of size V could represent more than 5 years of added, useful and reliable test results. For less rapid loss, size V could provide many years of reliable clinical information [6].

In this study we aimed, in a pragmatic clinical study, to investigate the information obtained from central static visual field assessment comparing size V versus size III targets using the Octopus 900 G programme in patients with advanced stage glaucoma. We hypothesise that, for many patients, we would obtain useful visual field results with size V to use as a new baseline for visual field status for subsequent comparison over follow-up, and further, comparative data of size III and V could be used to provide a recommendation for transfer from one test strategy to the other.

Materials and methods

Design

We undertook a prospective, exploratory, observational cross-section study. Institution ethical approval (Glaucoma Clinic New Delhi, India) was obtained and patients provided informed consent. We followed the STROBE checklist for reporting cross-sectional studies.

Recruitment

The study recruited patients with visual field loss due to advanced glaucoma and who met our inclusion criteria. Consecutive patients requiring visual fields were recruited from ophthalmology outpatient clinics between 2016-2017. Severe glaucoma was diagnosed according to the International Classification of Diseases (ICD) diagnostic code 9 (365.73) and 10 (7th digit “3”). Diagnostic code 365.73 represents the glaucoma stage code for severe, advanced and end-stage glaucoma consisting of “glaucomatous visual field abnormalities in both hemifields and/or loss

For peer review only - http://bmjopen.bmj.com/site/about/guidelines.xhtml

For peer review only

within 5 degrees of fixation in at least one hemifield" [11,12]. ICD version 9 codes were updated in 2015 to version 10 codes which consist of seven digits [13]. The first three indicate the code category and the last four provide added detail. Where the last digit is "3", this indicates severe glaucoma regardless of the type or cause of glaucoma. For example, H4010X3 represents unspecified open angle severe stage glaucoma.

Inclusion criteria

We included adult patients attending for visual field assessment with a diagnosis of severe or advanced glaucoma (stages 3-4), sufficient motor ability to sit at the perimeter unaided, ability to press the response button, sufficient cognitive ability to understand and follow instructions for performing the test, and willingness to undergo standard visual field assessments on the same day.

Exclusion criteria

We excluded patients with a diagnosis of stage 1-2 glaucoma, visual acuity worse than 1.0 logMAR, those unable to sit at the perimeter, those with unreliable visual fields, unable to follow instructions for performing the test or too ill to complete the full assessment.

Patient and public involvement

This study addresses a top research priority identified by patients and the public: "what is the most effective way of monitoring the progression of glaucoma?" (https://www.jla.nihr.ac.uk/).

Visual field assessment

Past studies have frequently used the 24-2 programme when testing visual fields in advanced glaucoma [3-5]. The Octopus 900 perimeter G programme has a physiology-based grid of 59 test locations within the central 30 degrees [14]. Locations are clustered more closely together centrally (2.8 degree spacing) with five central foveal locations and 17 test locations in the macular region. Locations are spaced further apart peripherally with emphasis on locations in nasal step regions and with more test locations nasally than temporally. There is no weighted analysis of test locations. Test locations are distributed in a pattern to follow retinal nerve fibre

For peer review only - http://bmjopen.bmj.com/site/about/guidelines.xhtml

bundles to facilitate the detection of glaucomatous visual field loss. Static visual field results were deemed unreliable if combined false negative and false positive responses exceeded 25%.

The study protocol consisted of visual field assessment with static G Octopus 900 perimetry using size III and size V targets (two tests per eye) on the same day. The order of assessment was randomised for size III versus size V. The G programme was run as standard with the size III target size (0.43 degrees in diameter) or with the size V target size (1.72 degrees in diameter).

Sample size

We studied two main outcomes: mean sensitivity and mean deviation.

For mean sensitivity sample size we assumed a difference of 3dB to be of clinical importance and hence to be detected. Wall et al. [5] reported lower and upper quartiles of 17.4dB and 24.5dB which are equivalent to a standard deviation of 3.55. Based on these figures, with pairwise t-test, significance level of 2.5% and power of 85%, this leads to a sample size of 15 eyes (one eye per patient).

For mean deviation, in the sample size we assume a difference of 10% decrease to be clinically relevant. Wall et al [4] showed a 15% decrease in their patients, while pooled standard deviation was 5.5dB7. With pairwise t-test, and significance level of 2.5%, and power of 85%, this leads to a sample size of 126 eyes (one eye per patient).

Statistical analysis

A direct comparison of results was made for static results using the statistical package SPSS version 25 (IBM SPSS Statistics, USA). The unit of analysis was per eye [15].

Glaucoma typically affects both eyes, thus each eye is not considered independent when considering analysis of visual field assessments. Assessments are usually reported for worst affected eye only. In this study we sought to evaluate visual field data available from size V versus size III perimetry. We had all the data from both eyes for many patients, hence we aimed to utilise all the data from all eyes as our primary analysis. For the purposes of sensitivity analysis, we also present results

For peer review only - http://bmjopen.bmj.com/site/about/guidelines.xhtml

from worst eyes only [15]. Worst eye was defined as the eye with greater mean deviation value on G1 static perimetry.

To evaluate normality of distribution of results from right and left eyes, a Kolmogorov-Smirnov test was used. Further we conducted a general linear model analysis for difference between eyes in mean sensitivity. The difference in sensitivity was the dependent variables and the eye (right versus left) was treated as random factor nested within patient. We also did analysis of the visual field measurement difference in the worse eye via a paired t-test.

To compare the results of the G programme when undertaken with size III and size V targets, variation was evaluated for:

- Mean sensitivity
- Mean deviation
- Standard loss variance

We further compared mean sensitivity and mean deviation values for each of the four quadrants of the visual field to determine if different quadrants showed comparable changes. Duration of assessments was also compared.

Scatterplots include linear regression lines and Loess curves. The linear regression line assesses strength of linear association between two continuous variables with r^2 =0.0 indicating lack of linear association (i.e. lack of correlation). We did not use errors-in-variables regression due to the fact this is an exploratory study. Correlation (r) is first assessed by its p-value (p) and then, if significant, the correlation is judged as none, small, medium or large if r is 0-0.1, 0.1-0.3, 0.3-0.5 or more than 0.5; i.e. when r^2 is 0-0.01, 0.01-0.09, 0.09-0.25 or more than 0.25 [16,17]. A non-directional p-value, i.e. two-sided alternative hypothesis, is used. The value of r^2 tells us how much variability is shared between variables. The Loess (locally estimated scatterplot smoothing) curve is a local nonlinear regression curve in which the fitting of each point is weighted towards the data nearest to that point. It makes no assumption about the association between variables and is used to visually observe the possible nature of the association between two continuous variables. It can be used as a modelling tool and provides a nonparametric regression that focuses on

For peer review only - http://bmjopen.bmj.com/site/about/guidelines.xhtml

the fitted curve [18]. The fitted points and related standard errors do respect a particular estimate but are estimated to the whole curve [19]. The default span was set to values ranging from 0.60-0.80 as a trade-off to ensure sufficient data for an accurate fit in order to reduce variance, and to avoid an over-smoothed regression in order to reduce bias. Along the Loess curve, the cross section between the x and y axis relating to the main point of inflection along the curve was used to indicate cut-off values between data represented along the x and y axes and therefore, for this study, used to calculate potential cut-off values for transfer of testing from size III to size V stimulus. The inflection point was taken to indicate at what value the relationship between III and V was possibly changing (as relevant when the linear relationship does not explain the association between III and V). The point of greatest curvature is suggested as a potential point of transitioning from size III to V as that is the point when the size III stimulus accelerates in its inability to detect change in visual field – relatively when compared to size V test. We report the inflection point as a range of values, as observed for the range of (α) 0.60-0.80.

In this paper all global indices are reported as positive numerical values, including mean sensitivity, mean deviation and standard loss variance.

Results

Primary analysis: All patients / eyes

Results are presented for 126 patients (170 eyes) with reliable visual field performance; 29 eyes at stage 3 and 141 at stage 4. A further 46 eyes were excluded because of unreliable visual field performance or visual field results graded at stage 2 or better. The worst affected eye was right in 64 (50.8%) and left in 62 (49.2%). Mean age at time of testing was 55.86 years (SD 15.15; 58 [range 13-83]). Figure 1 displays one example of size III versus size V results for one patient.

Distribution of data

Visual field assessment data was tested to be normally distributed (Kolmogorov-Smirnov test). Further, general linear model analysis with random effect being the subject and fixed effect being 1) eye laterality (right versus left eye) or 2) worst

For peer review only - http://bmjopen.bmj.com/site/about/guidelines.xhtml

Table 1: Differences in visual field parameters for target size – all eyes

	**G1, size III**		**G1, size V**		**Difference**				
	Mean	SD	Mean	SD	Mean	SD	General linear mixed model (subject)	Correlation significance	Bonferroni correction
MS	6.94	5.58	12.98	7.77	-6.04	4.18	0.0001	0.005	0.0002
MD	20.04	5.67	19.22	7.74	0.82	4.15	0.023	0.007	0.046
SLV	5.89	2.29	7.69	2.78	-1.80	2.60	0.0001	0.0001	*
DD	17.13	7.86	14.76	9.47	2.37	5.46	0.0001	0.009	*
MSnasalsup	5.31	5.48	10.48	8.78	-5.17	5.21	0.0001	0.0001	*
MSnasalinf	7.70	7.48	14.40	9.74	-6.70	5.33	0.0001	0.004	*
MStempsup	6.27	5.78	12.05	8.68	-5.77	5.42	0.0001	0.114	*
MStempinf	8.23	7.59	14.68	10.12	-6.45	5.37	0.0001	0.005	*
MDnasalsup	21.29	5.55	21.13	8.70	0.16	5.16	0.662	0.001	*
MDnasalinf	19.59	7.58	18.28	9.77	1.31	5.31	0.010	0.005	*

For peer review only - http://bmjopen.bmj.com/site/about/guidelines.xhtml

MDtempsup	20.25	5.86	19.58	8.60	0.67	5.37	0.119	0.123	*
MDtempinf	19.06	7.61	18.00	10.08	1.06	5.37	0.018	0.006	*
Duration	3.32	1.07	6.40	1.43	-3.07	1.66	0.0001	0.015	*

MS – mean sensitivity; MD – mean deviation; SLV – standard loss variance; DD – diffuse defect; Temp – temporal; Sup – superior; Inf – inferior

* Not corrected as exploratory analysis only

Table 2: Differences in visual field parameters for target size – worst eyes

	**G1, size III**		**G1, size V**		**Difference**			
	Mean	SD	Mean	SD	Mean	SD	Paired t test	Bonferroni correction
**MS**	6.31	5.57	12.24	7.67	-5.93	4.43	0.0001	0.0002
**MD**	20.72	5.63	20.00	7.59	0.72	4.39	0.072	0.144
**SLV**	5.73	2.34	7.67	2.70	-1.94	2.59	0.0001	*
**DD**	18.13	7.84	15.58	9.44	2.54	5.78	0.0001	*

For peer review only - http://bmjopen.bmj.com/site/about/guidelines.xhtml

**MSnasalsup**	4.80	5.38	9.92	8.57	-5.11	5.47	0.0001	*
**MSnasalinf**	7.00	7.52	13.64	9.61	-6.64	5.36	0.0001	*
**MStempsup**	5.73	5.85	11.26	8.74	-5.52	5.80	0.0001	*
**MStempinf**	7.49	7.44	13.86	10.06	-6.37	5.65	0.0001	*
**MDnasalsup**	21.88	5.42	21.75	8.46	0.12	5.42	0.801	*
**MDnasalinf**	20.34	7.57	19.08	9.60	1.27	5.34	0.01	*
**MDtempsup**	20.85	5.93	20.42	8.62	0.43	5.73	0.407	*
**MDtempinf**	19.85	7.46	18.86	9.99	0.99	5.64	0.053	*
**Duration**	3.31	1.07	6.36	1.37	-3.05	1.65	0.0001	*

MS – mean sensitivity; MD – mean deviation; SLV – standard loss variance; DD – diffuse defect; Temp – temporal; Sup – superior; Inf – inferior

* Not corrected as exploratory analysis only

For peer review only

versus better eye, with respect to mean sensitivity, showed no significant difference between size V versus size III data (p=0.888 and p=0.644 respectively).

Size III versus size V differences

Results are outlined in table 1 for differences, and significance, for mean sensitivity, mean deviation and standard loss variance, along with differences across quadrants plus test duration. Differences for mean sensitivity between sizes III and V were about 6 decibels overall and 5-7 decibels across quadrants with greatest increases in inferior quadrants. A strong linear relationship and large correlation was found between mean sensitivity for size III and V stimuli (Figure 2: p=0.0001, r^2 0.731, 0.855). Using the Loess curve to consider differences between size III and V stimuli, the point of greatest inflection (visualised on the scatterplot with value taken from y axis) is approximately 4-6 decibels for size III stimulus.

Global mean deviation was significantly lower for size V but not clinically significant with significant differences only in inferior quadrants. A strong linear relationship and large correlation was found between mean deviation for size III and V stimuli (Figure 2: p=0.0001, r^2 0.735, -0.857. Using the Loess curve to consider differences between size III and V stimuli, the point of greatest inflection is approximately 22-24 decibels for size III stimulus. Diffuse defect was lower and standard loss variance was higher for size V (see Supplementary Figure 1) representing more visual field responses with the size V target. Test duration increased for size V but was likely due to more patient responses being obtained due to presence of more visible targets.

Sensitivity analysis: Worst eyes only

Taking only the worst eye for each patient, results are presented for 126 patients (126 eyes) with 18 eyes at stage 3 and 108 at stage 4. The worst eye was right in 64 (50.8%) and left in 62 (49.2%).

Size III versus size V differences

Results are outlined in table 2 for differences, and significance, for mean sensitivity, mean deviation and standard loss variance, along with differences across quadrants plus test duration. Differences for mean sensitivity between size III and V were about 6 decibels overall and 5-7 decibels across quadrants with greatest increases in

For peer review only - http://bmjopen.bmj.com/site/about/guidelines.xhtml

inferior quadrants. A strong linear relationship and large correlation was found between mean sensitivity for size III and V stimuli (Figure 3: p=0.0001, r^2 0.678, 0.823). Using the Loess curve to consider differences between size III and V stimuli, the point of greatest inflection (visualised on the scatterplot with value taken from y axis) is approximately 3-5 decibels for size III stimulus.

Global mean deviation was significantly lower for size V but not clinically significant (Figure 3) with significant differences only in inferior quadrants. A strong linear relationship and large correlation was found between mean deviation for size III and V stimuli (Figure 3: p=0.0001, r^2 0.687, -0.829). Using the Loess curve to consider differences between size III and V stimuli, the point of greatest inflection is approximately 22-24 decibels for size III stimulus. Diffuse defect was lower and standard loss variance was higher for size V (see Supplementary Figure 2) representing more visual field responses. Test duration increased for size V but was likely due to more patient responses being obtained due to presence of more visible targets.

Discussion

Detection of visual field loss in glaucoma and other optic neuropathies is well reported with discussion of early detection methods, exploring visual field defects with steep border slopes and for conversion of disease/progression [1,20]. Studies have mostly used the 24-2 central static threshold programme [3-5]. In this study we sought to explore the use of size V versus size III targets using the Octopus 900 G programme to evaluate detection of absolute versus relative visual field loss in advanced (stages 3-4) glaucoma. This was a pragmatic clinical study in which we primarily wished to evaluate how much additional useful information could be obtained from a switch to stimulus size V at a stage of moderate to severe glaucoma when visual field results had reached a level of loss with stimulus size III, such that meaningful information could no longer be extracted from the results to guide clinical decision making.

One recent study sought to gather patient perspectives and reliability based on clinical grounds whilst comparing size III and V stimulus sizes using a 24-2 test

For peer review only - http://bmjopen.bmj.com/site/about/guidelines.xhtml

For peer review only

programme [21]. They concluded that adjustment of testing to use of size V increased their patient cooperation and was a valid option when attempting to gather more reliable perimetry results. From our clinical perspective, we found the results using size V to provide more visual field data to help inform clinical decision making and a grounding towards future disease monitoring.

The mean sensitivity provides a single global indicator value for the overall visual field sensitivity. We found the mean sensitivity value to increase overall by about 6 decibels for size V versus size III but with a difference of about 10 decibels (in keeping with that expected by the change in stimulus size) at mean sensitivities of 10-30 decibels. This indicates relative visual field loss for size V stimulus compared to the indication of absolute defects for size III stimulus and is indicative of the relationship between stimulus size and luminance [21]. Many perimeter parameters are such that at maximum luminance, the size III target has a calibrated range of 2-44 decibels and the size V target has a range of 10-54 decibels (10 decibel difference). As expected, the larger stimulus size increases sensitivity and this is particularly so at greater eccentricities [6]. In clinical studies this is shown as the effective dynamic range of size V being about one log unit greater than size III [3]. Gardiner and colleagues confirmed size V resulted in higher sensitivity at the same location, providing more reliable and less variable estimates of sensitivity in damaged visual fields. Their higher sensitivity was on average 5.6 decibels – similar to our average of 6 decibels and to the average of 6 and 7.6 decibels reported by Morgan et al [21] and Choplin et al [8], respectively.

The mean deviation provides a single global indicator value for the overall visual field sensitivity in comparison to an age-matched normal visual field. We found a slight but clinically insignificant reduction in mean deviation for size V versus III. The association between size III and V targets has been reported as approximately linear to a sensitivity of about 20 decibels. Size V had a greater effective dynamic range with eight discriminable steps for progression – about twice as many as with size III [22].

A second objective of our study was to consider indication levels for a transfer from size III to size V for static perimetry. In our analysis, we used the Loess curve to compare differences between size III and V stimuli. The Loess curve does not

For peer review only - http://bmjopen.bmj.com/site/about/guidelines.xhtml

provide confidence values. We found the point of inflection to be within an overall range of approximately 4-6 decibels for size III stimulus on mean sensitivity and 22-24 decibels on mean deviation. Arguably, the inflection point might be considered the latest point at which to switch from size II to size V. A more sensible approach would be to switch before severe damage to the visual field occurs to allow progression to be more reliably tracked with size V over, potentially, a longer period of time. We therefore provide a clinical recommendation that at a mean sensitivity level of 10 decibels (4 decibels above the higher inflection estimate) and/or at a mean deviation level of 18 decibels (4 decibels below the lower inflection estimate) on a visual field using size III, a switch may be made to using size V stimuli for subsequent visual field assessment.

Mean deviation reduced more for inferior visual field quadrants. Our discrepancy between superior and inferior visual field cannot be explained by the number of test locations as test location numbers are equal for the superior and inferior visual field. The greater reduction in mean deviation for inferior visual field may be explained by the presence of better inferior visual field compared to the extent of superior visual field loss. In both primary open and closed angle glaucoma, visual field damage has often been reported as being more severe in the superior visual field than inferiorly [23].

Variability for size V targets has been reported as being substantially lower than for size III [3]. However, in later studies, Wall and colleagues [4,5] did not find a significant reduction in size V variability compared to size III. This was partly explained by the use of size V allowing patients to detect more targets than with the smaller size III – 0 decibel responses using size III become detectable decibel responses with size V. Thus, as patients can see more, the extra responses add to variability. This was not apparent in cases with most severe visual field loss with mean sensitivities close to 0 decibel but emerged from a mean sensitivity of 10 decibels or higher, i.e. when sufficient visual field was discernible. We found mean deviation to be higher for size III generally up to 20-25 decibels at which level mean deviation for size V was higher which likely reflects the greater extent of visual field seen with size V than size III. This effect disappeared for very poor visual fields, even with size V. Our study only included stage 3-4 advanced glaucoma with cases where there was substantial visual field loss to the extent that perimetry with size III

For peer review only - http://bmjopen.bmj.com/site/about/guidelines.xhtml

no longer provided adequate information to facilitate monitoring of the condition for progression/response to treatment. Changing to size V allowed us to further evaluate visual fields with more visual field information being obtained.

The standard loss variance provides a single global indicator value for the extent of localised visual field loss that is present. Our results indicate higher standard loss variance measures with size V likely due to greater visual field responses being obtained with the larger size target. Further, and not surprising, we found longer test durations for size V compared to size III and in keeping with other studies [21]. This reflects the great visual response to the larger size V target. Thus, the more targets than can be detected and responded to, the longer the test time will be.

We addressed a methodological issue with this paper. Many studies reporting visual field analysis present data from one eye: often the worst eye. We evaluated the distribution of data for all eyes in this study as our primary analysis. We subsequently ran a sensitivity analysis of worst eyes only to determine where significance lies when only focussing on the worst eye and where the sample size is smaller. We found no differences in distribution for right versus left eyes or for worst versus better eyes except for mean deviation which showed more significance for primary analysis and which likely reflects the larger sample size for primary analysis (all eyes). Such evaluation of distribution is essential if wishing to combine results from all eyes in reporting data. Clearly analysis and reporting of data is important in the context of the type of study. Reporting intervention outcomes often necessitates a determination of independent effect. Hence reporting data from one eye per subject is often appropriate where the condition affects both eyes. However, in studies of assessment comparisons (such as this present study) the question being asked is about the ability of each assessment/test to detect the same defect and that question is as important for each eye individually (as two independent comparisons) as for the patients individually (as a single comparison). We have reported our methods and results in establishing the distribution of data and our subsequent decision to combine results versus solely presenting worst eye data only. As seen from our results across tables 1 and 2, values were highly comparable for all eyes versus worst eyes only.

Limitations

For peer review only - http://bmjopen.bmj.com/site/about/guidelines.xhtml

Our study is limited by a number of factors. We report visual field results but did not collect visual acuity or fundus imaging for this study. We also did not formally record patient preference for visual field testing with size III or V. Further, we did not evaluate test reliability by accounting for false positive and false negative responses, although excessive responses were exclusion criteria for our study.

Conclusions

Use of size V versus III in standard automated perimetry for advanced stages 3-4 in glaucoma permits further visual field to be plotted in cases where the visual fields with size III no longer provides sufficient information to appropriately monitor the disease. Mean sensitivity, standard loss variance and test duration increased in accordance with the extra visual field responses obtained using size V. We do not infer that automated perimetry with size V is better than with size III. Size V is easier to detect and, thus, from a pragmatic clinical view, a switch from size III to V in patients with advanced disease was a logical alteration to the visual field assessment protocol. We recommend considering a change to size V stimulus for advanced glaucoma at stages 3-4 when, with size III, the mean sensitivity reaches 10 decibels and/or mean deviation reaches 18 decibels, to facilitate continued assessment of visual fields as part of the clinical monitoring of the condition.

References

1. Bua S, Supuran CT. (2019) Diagnostic markers for glaucoma: a patent and literature review (2013-2019). Expert Opin Ther Pat. doi: 10.1080/13543776.2019.1667336
2. Tomairek RH, Aboud SA, Hassan M, Mohamed AH. (2019) Studying the role of 10-2 visual field test in different stages of glaucoma. Eur J Ophthalmol. 1-8: DOI: 10.1177/1120672119836904
3. Wall M, Brito CF, Woodward KR, Doyle CK, Kardon RH, Johnson CA. (2008) Total deviation probability plots for stimulus size V perimetry. Arch Ophthalmol. 126: 473-479

For peer review only - http://bmjopen.bmj.com/site/about/guidelines.xhtml

4. Wall M, Doyle CK, Zamba KD, Artes P, Johnson CA. (2013) The repeatability of mean defect with size III and size V standard automated perimetry. Invest Ophthalmol Vis Sci. 54: 1345-1351
5. Wall M, Doyle CK, Eden T, Zamba KD, Johnson CA. (2013) Size threshold perimetry performs as well as conventional automated perimetry with stimulus sizes III, V and VI for glaucomatous loss. Invest Ophthalmol Vis Sci. 54: 3975-3983
6. Gardiner SK, Demirel S, Goren D, Mansberger SL, Swanson WH. (2015) The effect of stimulus size on the reliable stimulus range of perimetry. Tran Vis Sci Tech. 4(2): 10, http://tvstjournal.org/doi/full/10. doi:10.1167/tvst.4.2.10
7. Swanson WH. (2013) Stimulus size for perimetry in patients with glaucoma. Research highlight. Invest Ophthalmol Vis Sci. 54(6):3984
8. Choplin NT, Sherwood MB, Spaeth GL. (1990) The effect of stimulus size on the measured threshold values in automated perimetry. Ophthalmol. 97:371–374.
9. Gilpin L, Stewart W, Hunt H, Broom C. (1990) Threshold variability using different Goldmann stimulus sizes. Acta Ophthalmol. 68:674–676.
10. Wall M, Kutzko K, Chauhan B. (1997) Variability in patients with glaucomatous visual field damage is reduced using size V stimuli. Invest Ophthalmol Vis Sci. 38:426–435.
11. WuDunn D. (2015) Monitoring patients with advanced glaucoma. Glaucoma Today. September/October: 42-43
12. Fellman RL, Mattox CG, Ross KM, Vicchrilli S. (2011) Know the new glaucoma staging codes. Eyenet. October: 65-66
13. https://icd.who.int/browse10/2010/en#/H40-H42 [last accessed 12th May 2020]
14. Racette L, Fischer M, Bebie H, Hollo G, Johnson CA, Matsumoto C. (2016) Visual field digest. A guide to perimetry and the Octopus perimeter. 6th edition. Haag Streit AG, Switzerland
15. Bunce C, Patel KV, Xing W, Freemantle N, Dore CJ, (2014) Ophthalmic Statistics Group. Ophthalmic statistics note 1: unit of analysis. Br J Ophthalmol. 98: 408-12

For peer review only - http://bmjopen.bmj.com/site/about/guidelines.xhtml

16. Illowsky B, Dean S. (2013) Introductory Statistics. OpenStax, Houston, Texas. https://openstax.org/books/introductory-statistics/pages/12-4-testing-the-significance-of-the-correlation-coefficient [accessed 29th May 2020]
17. Cohen J. (1988) Statistical power analysis for the behavioural sciences. Routledge. ISBN 978-1-134-74270-7
18. Cleveland WS, Grosse E, Shyu WM. (1992) Local regression models. Chapter 8. Statistical Models in S. Editors: Chambers JM and Hastie TJ, Wadsworth & Brooks/Cole
19. https://www.statsdirect.com/help/nonparametric_methods/loess.htm [accessed 29th May 2020]
20. Peters D, Bengtsson B, Heijl A. (2015) Threat to fixation at diagnosis and lifetime risk of visual impairment in open-angle glaucoma. Ophthalmol. 122:1034-9
21. Morgan AM, Mazzoli LS, Caixeta-Umbelino C, Kasahara N. (2019). Expediency of the Automated Perimetry Using the Goldmann V Stimulus Size in Visually Impaired Patients with Glaucoma. Ophthalmol Ther, 8(2), 305–311
22. Wall M, Woodward KR, Doyle CK, Zamba G. (2010) The effective dynamic ranges of standard automated perimetry sizes III and V and motion and matrix perimetry. Arch Ophthalmol. 128: 570-576
23. Yousefi S, Sakai H, Murata H, Fujino Y, Garway-Heath D, Weinreb R, Asaoka R. (2018) Asymmetric Patterns of Visual Field Defect in Primary Open-Angle and Primary Angle-Closure Glaucoma. Invest Ophthalmol Vis Sci. 59:1279-1287

For peer review only

Figure legends

Figure 1: Illustrative results of static size III versus size V perimetry

A – G1 static perimetry with size III, left eye

B – G1 static perimetry with size V, left eye

Static perimetry (using size III) results shows a severely restricted visual field whilst static perimetry (using size V) results display better temporal visual field and inferonasal visual field.

Figure 2: Mean sensitivity and mean deviation comparisons – Primary analysis of all eyes.

A – Mean sensitivity

For peer review only - http://bmjopen.bmj.com/site/about/guidelines.xhtml

Straight line is the linear regression line; curved line is the Loess regression curve.

Comparisons generally show clustering where mean sensitivity is close to 0 decibels, i.e. worst visual field. There is a “floor” effect with size III, yielding many zeros, while size V gives, for most, larger values.

The linear regression line and Loess regression curve show large correlation for size V (r 0.855, r^2 0.731, $p<0.0001$). The Loess point of inflection (value taken from the y axis) is approximately 4-6 decibels.

B – Mean deviation

Straight line is the linear regression line; curved line is the Loess regression curve.

The linear regression line and Loess regression curve show large correlation for size V (r -0.857, r^2 0.735, $p<0.0001$). The Loess point of inflection (value taken from the y axis) is approximately 22-24 decibels.

Figure 3: Mean sensitivity and mean deviation comparisons – Sensitivity analysis of worst eyes.

A – Mean sensitivity

Straight line is the linear regression line; curved line is the Loess regression curve.

Comparisons generally show clustering where mean sensitivity is close to 0 decibels, i.e. worst visual field. There is a “floor” effect with size III, yielding many zeros, while size V gives, for most, larger values.

The linear regression line and Loess regression curve show large correlation for size V (r 0.823, r^2 0.678, $p<0.0001$). The Loess point of inflection (value taken from the y axis) is approximately 3-5 decibels.

B – Mean deviation

Straight line is the linear regression line; curved line is the Loess regression curve.

The linear regression line and Loess regression curve show large correlation for size V (r -0.829, r^2 0.687, $p<0.0001$). The Loess point of inflection (value taken from the y axis) is approximately 22-24 decibels.

For peer review only

Supplementary Figure 1: Standard loss variance, diffuse defect and test duration comparisons – Primary analysis of all eyes.

A: Standard loss variance

B: Diffuse defect

C: Test duration

Straight line is the linear regression line; curved line is the Loess regression curve. Loss variance is variable for sizes III and V but with higher loss variance for size V at 8-12 decibels. The linear regression line and Loess regression curve show medium to large correlation for size V (r 0.507, r^2 0.257, p<0.0001). The Loess point of inflection (value taken from the y axis) is approximately 5-7 decibels (1a).
Diffuse defect is higher for size III at 10-20 decibels but higher for size V at 25-30 decibels. The linear regression line and Loess regression curve show large correlation for size V (r 0.820, r^2 0.672, p<0.0001). The Loess point of inflection (value taken from the y axis) is approximately 23-25 decibels (1b).
Test duration increased for size V versus size III. The linear regression line and Loess regression curve show small correlation for size V (r 0.200, r^2 0.040, p<0.009). The Loess point of inflection (value taken from the y axis) is approximately 3-5 minutes (1c).

Supplementary Figure 2: Standard loss variance, diffuse defect and test duration comparisons – Sensitivity analysis of worst eyes.

A: Standard loss variance

B: Diffuse defect

C: Test duration

Straight line is the linear regression line; curved line is the Loess regression curve. Loss variance is variable for sizes III and V but with higher loss variance for size V at 8-12 decibels. The linear regression line and Loess regression curve show medium to large correlation for size V (r 0.510, r^2 0.260, p<0.0001). The Loess point of inflection (value taken from the y axis) is approximately 4-6 decibels (2a).
Diffuse defect is higher for size III at 10-20 decibels but higher for size V at 25-30 decibels. The linear regression line and Loess regression curve show large

For peer review only - http://bmjopen.bmj.com/site/about/guidelines.xhtml

correlation for size V (r 0.797, r^2 0.635, p<0.0001). The Loess point of inflection (value taken from the y axis) is approximately 21-23 decibels (2b).

Test duration increased for size V versus size III. The linear regression line and Loess regression curve show small correlation for size V (r 0.190, r^2 0.036, p<0.035). The Loess point of inflection (value taken from the y axis) is approximately 3-5 minutes (2c).

For peer review only

For peer review only - http://bmjopen.bmj.com/site/about/guidelines.xhtml

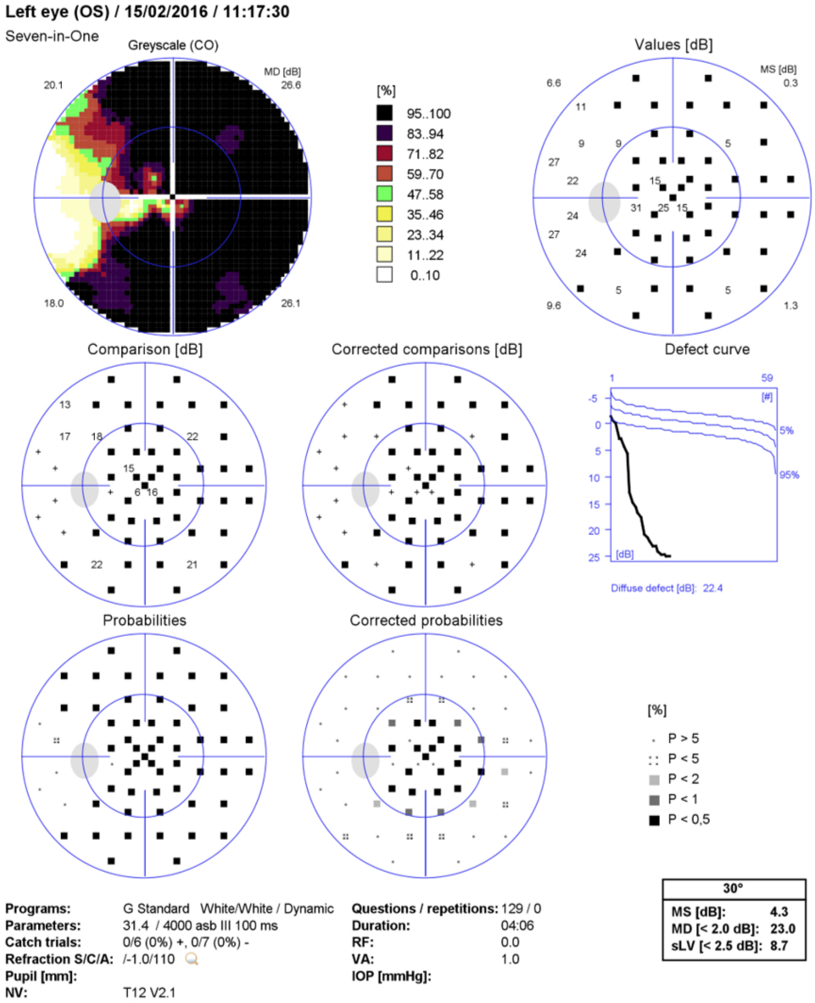

179x182mm (150 x 150 DPI)

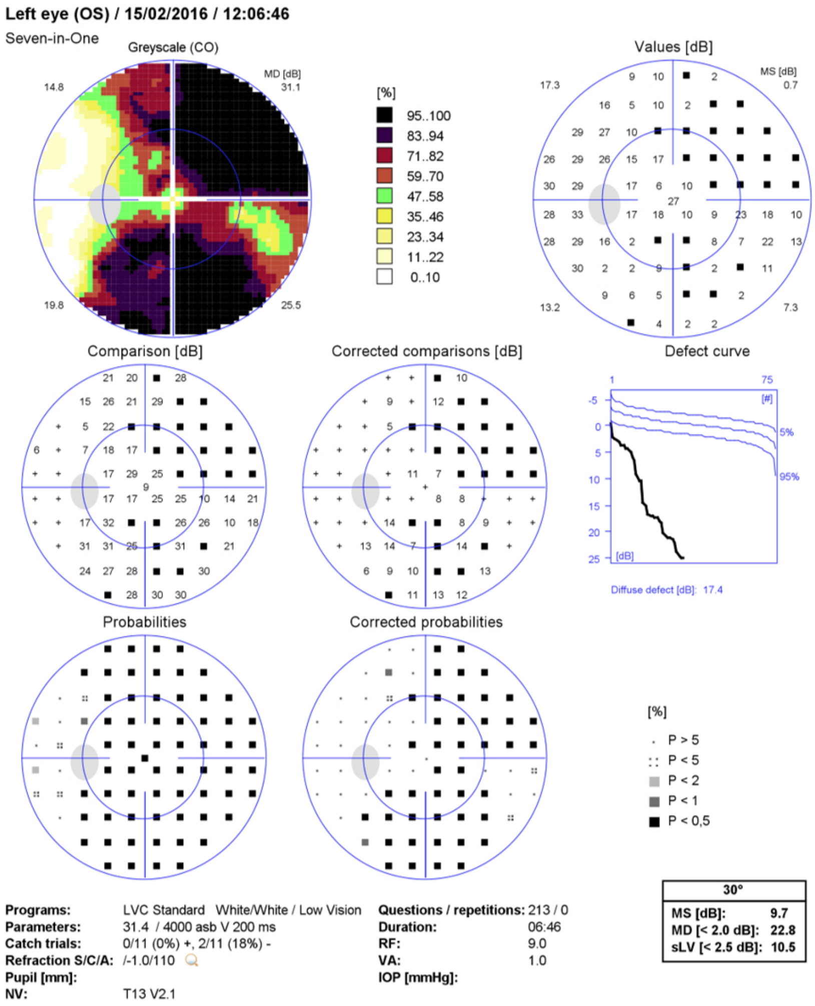

179x184mm (150 x 150 DPI)

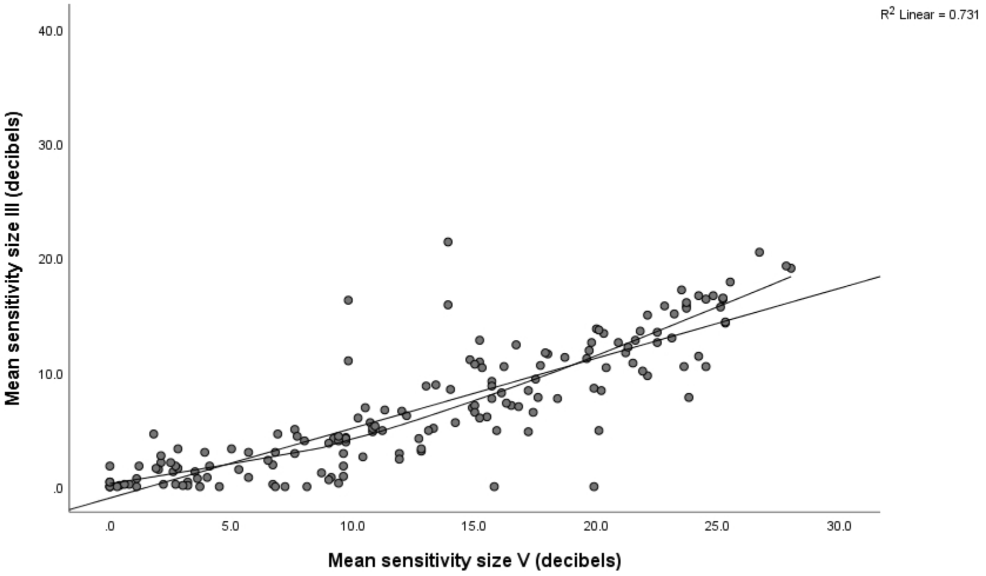

225x132mm (96 x 96 DPI)

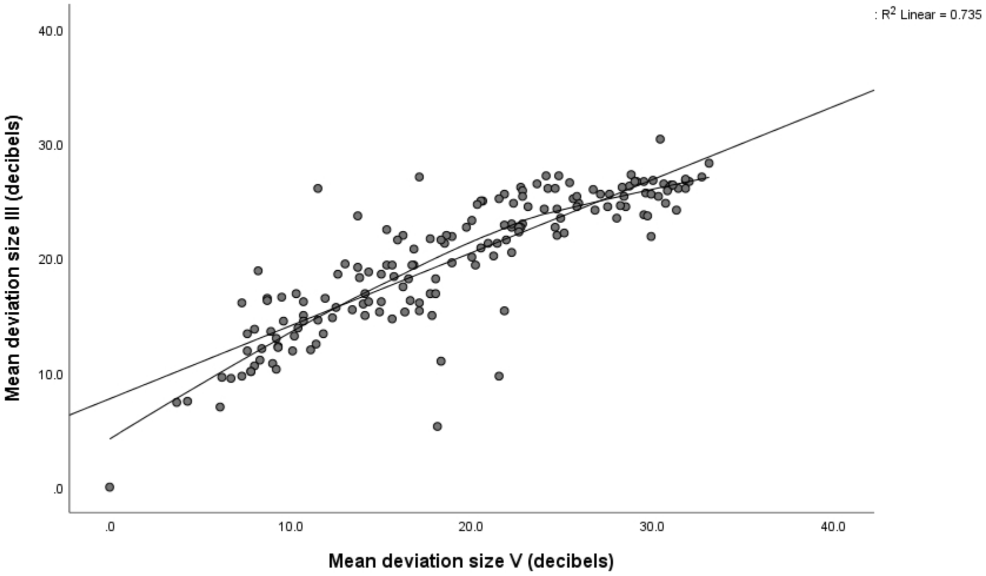

224x139mm (96 x 96 DPI)

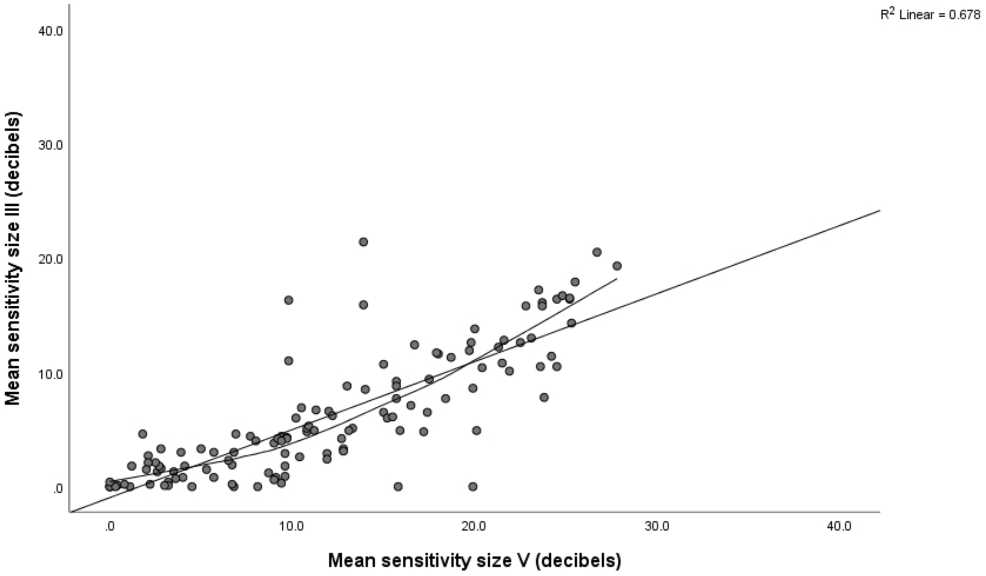

225x132mm (96 x 96 DPI)

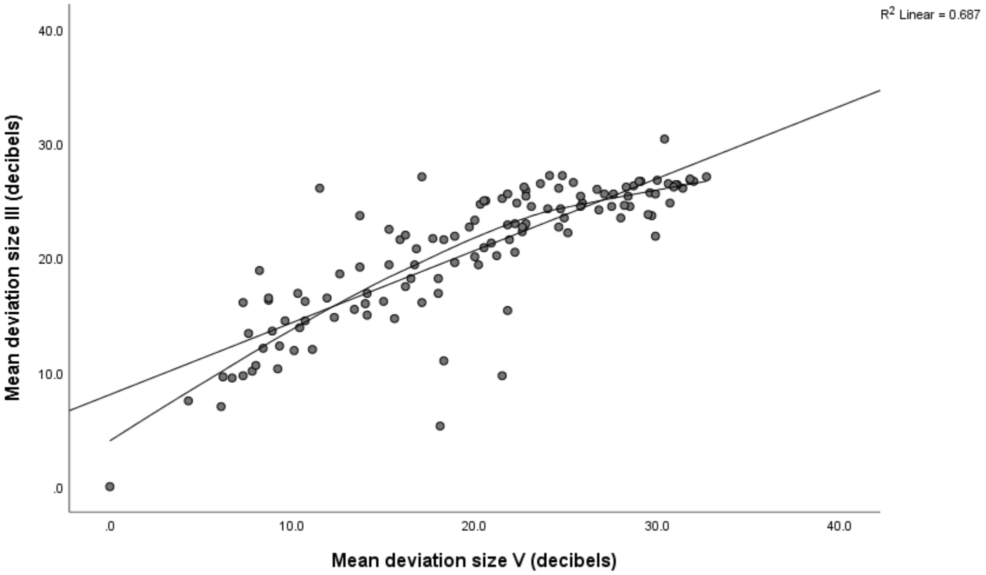

225x132mm (96 x 96 DPI)

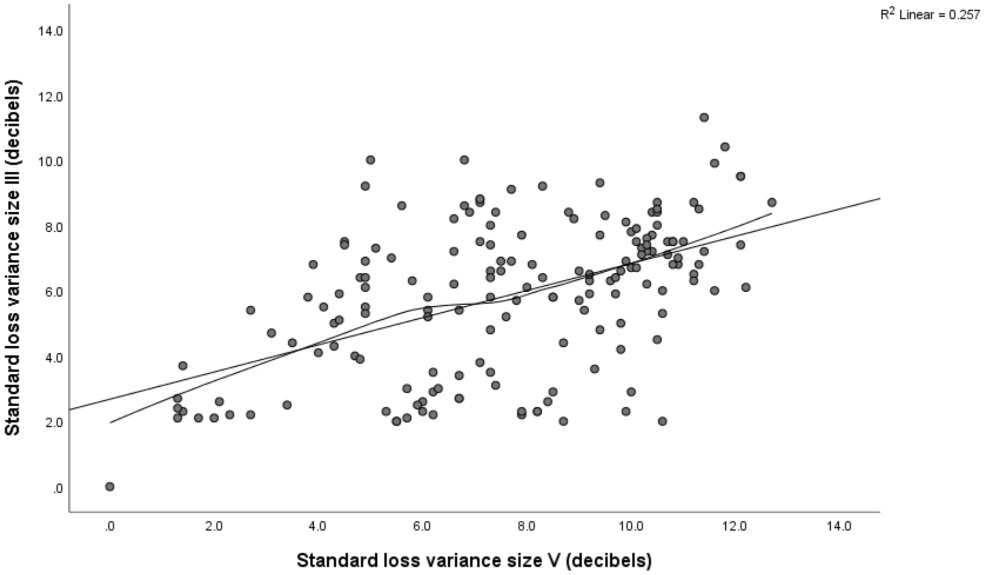

225x132mm (96 x 96 DPI)

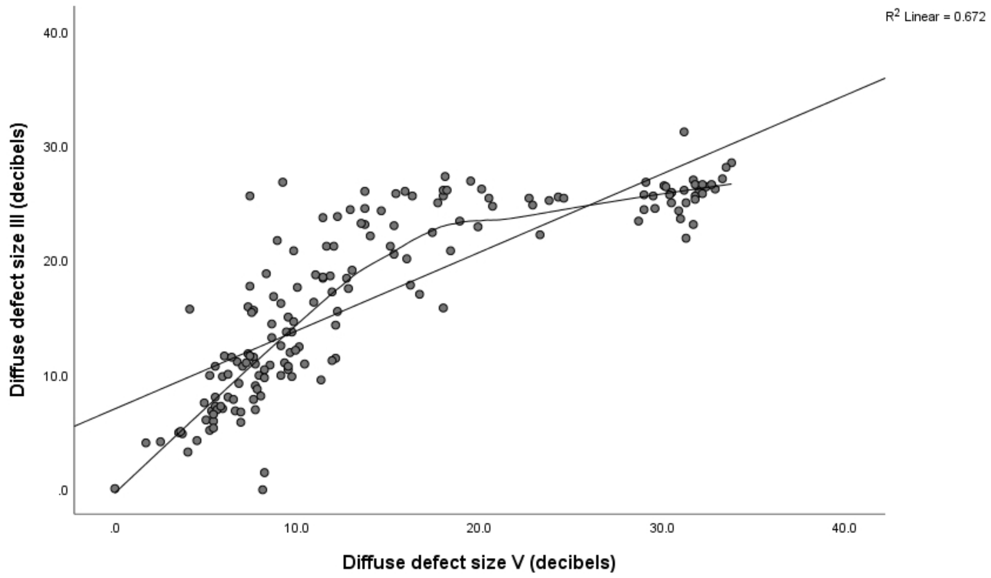

225x132mm (96 x 96 DPI)

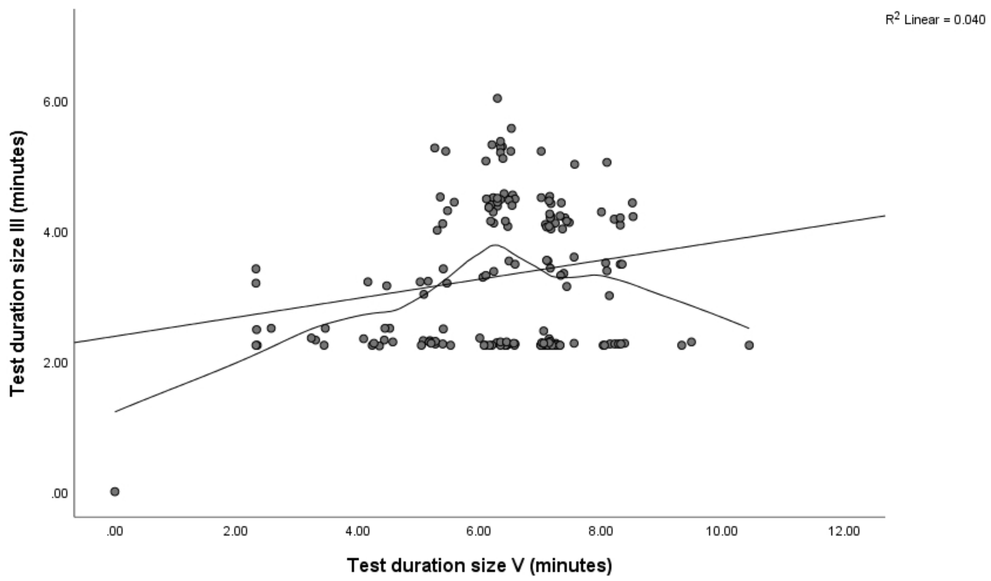

225x132mm (96 x 96 DPI)

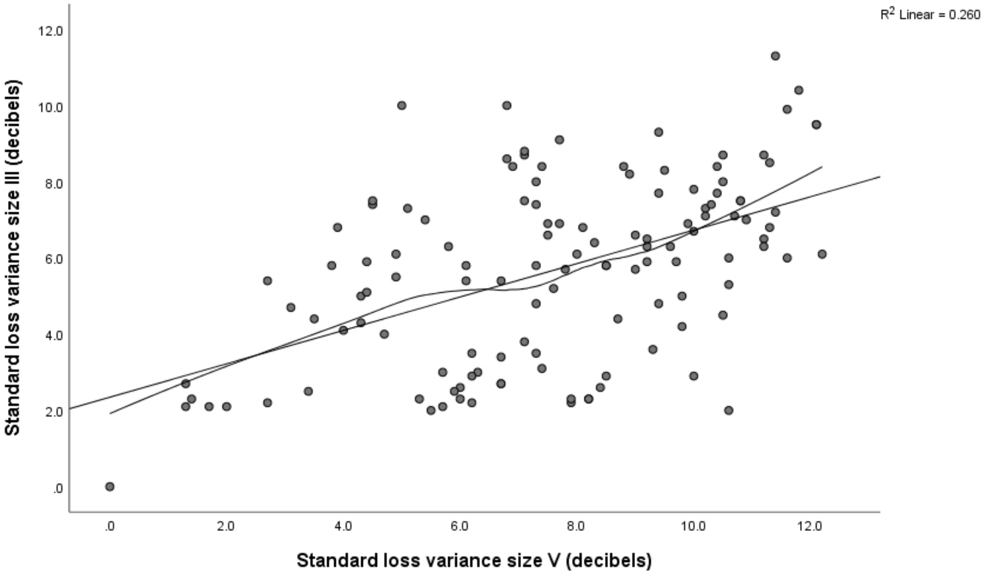

225x132mm (96 x 96 DPI)

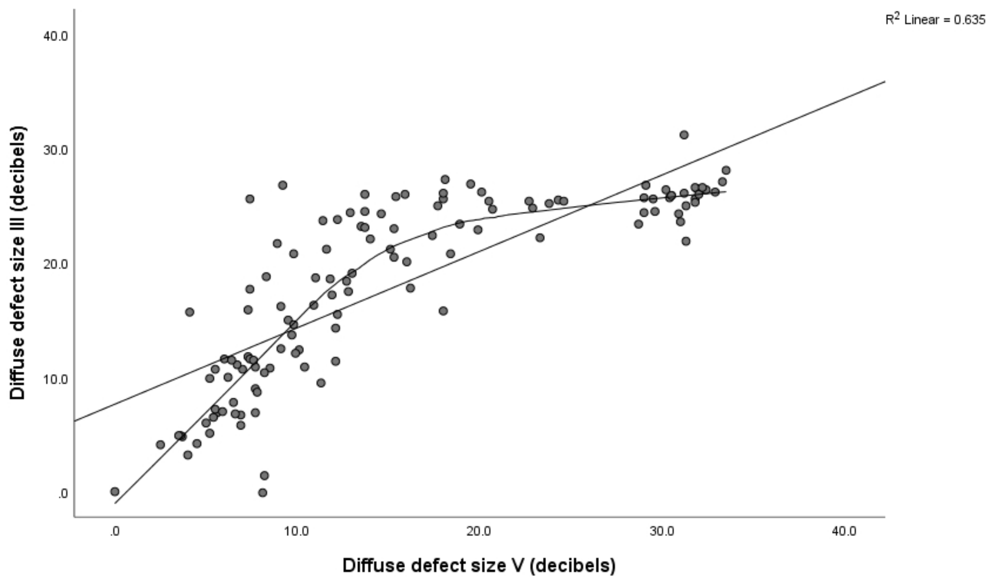

225x132mm (96 x 96 DPI)

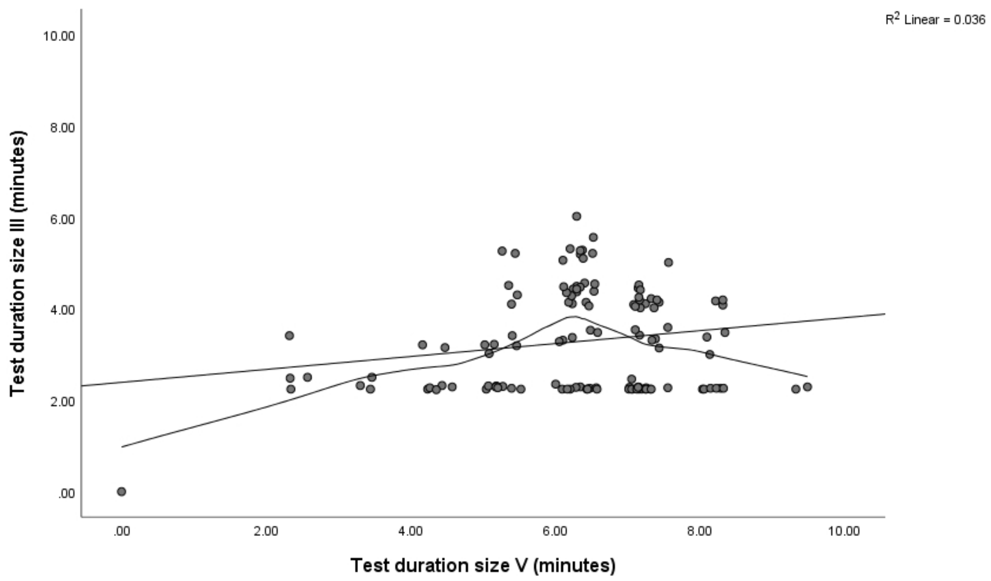

225x132mm (96 x 96 DPI)

STROBE Statement—Checklist of items that should be included in reports of ***cross-sectional studies***

	Item No	Recommendation	Page No
**Title and abstract**	1	(*a*) Indicate the study's design with a commonly used term in the title or the abstract	1
		(*b*) Provide in the abstract an informative and balanced summary of what was done and what was found	2
**Introduction**			
Background/rationale	2	Explain the scientific background and rationale for the investigation being reported	3-5
Objectives	3	State specific objectives, including any prespecified hypotheses	5
**Methods**			
Study design	4	Present key elements of study design early in the paper	5
Setting	5	Describe the setting, locations, and relevant dates, including periods of recruitment, exposure, follow-up, and data collection	5-9
Participants	6	(*a*) Give the eligibility criteria, and the sources and methods of selection of participants	5-7
Variables	7	Clearly define all outcomes, exposures, predictors, potential confounders, and effect modifiers. Give diagnostic criteria, if applicable	5-7
Data sources/ measurement	8*	For each variable of interest, give sources of data and details of methods of assessment (measurement). Describe comparability of assessment methods if there is more than one group	5-7
Bias	9	Describe any efforts to address potential sources of bias	7-9
Study size	10	Explain how the study size was arrived at	7-9
Quantitative variables	11	Explain how quantitative variables were handled in the analyses. If applicable, describe which groupings were chosen and why	7-9
Statistical methods	12	(*a*) Describe all statistical methods, including those used to control for confounding	7-9
		(*b*) Describe any methods used to examine subgroups and interactions	7-9
		(*c*) Explain how missing data were addressed	7-9
		(*d*) If applicable, describe analytical methods taking account of sampling strategy	
		(*e*) Describe any sensitivity analyses	
**Results**			
Participants	13*	(a) Report numbers of individuals at each stage of study—eg numbers potentially eligible, examined for eligibility, confirmed eligible, included in the study, completing follow-up, and analysed	9
		(b) Give reasons for non-participation at each stage	9
		(c) Consider use of a flow diagram	
Descriptive data	14*	(a) Give characteristics of study participants (eg demographic, clinical, social) and information on exposures and potential confounders	9-13
		(b) Indicate number of participants with missing data for each variable of interest	9
Outcome data	15*	Report numbers of outcome events or summary measures	9-13
Main results	16	(*a*) Give unadjusted estimates and, if applicable, confounder-adjusted estimates and their precision (eg, 95% confidence interval). Make clear which confounders were adjusted for and why they were included	10-13

For peer review only

		(*b*) Report category boundaries when continuous variables were categorized	10-13
		(*c*) If relevant, consider translating estimates of relative risk into absolute risk for a meaningful time period	
Other analyses	17	Report other analyses done—eg analyses of subgroups and interactions, and sensitivity analyses	13-14
**Discussion**			
Key results	18	Summarise key results with reference to study objectives	14
Limitations	19	Discuss limitations of the study, taking into account sources of potential bias or imprecision. Discuss both direction and magnitude of any potential bias	18
Interpretation	20	Give a cautious overall interpretation of results considering objectives, limitations, multiplicity of analyses, results from similar studies, and other relevant evidence	14-17
Generalisability	21	Discuss the generalisability (external validity) of the study results	14-17
**Other information**			
Funding	22	Give the source of funding and the role of the funders for the present study and, if applicable, for the original study on which the present article is based	3

*Give information separately for exposed and unexposed groups.

Note: An Explanation and Elaboration article discusses each checklist item and gives methodological background and published examples of transparent reporting. The STROBE checklist is best used in conjunction with this article (freely available on the Web sites of PLoS Medicine at http://www.plosmedicine.org/, Annals of Internal Medicine at http://www.annals.org/, and Epidemiology at http://www.epidem.com/). Information on the STROBE Initiative is available at www.strobe-statement.org.

For peer review only

Standard automated perimetry using size III and size V stimuli in advanced stage glaucoma: an observational cross-sectional comparative study.

Journal:	*BMJ Open*
Manuscript ID	bmjopen-2020-046124.R1
Article Type:	Original research
Date Submitted by the Author:	27-Jul-2021
Complete List of Authors:	Sood, Devindra; Glaucoma Clinic New Delhi, Ophthalmology<br>Czanner, Gabriella; Liverpool John Moores University, Statistics and data science<br>Somerville, Tobi; University of Liverpool Faculty of Health and Life Sciences, Eye and Vision Science<br>Sood, Ishaana; SK Glaucoma Care Foundation, Ophthalmology<br>Rowe, Fiona; University of Liverpool Faculty of Health and Life Sciences, Institute of Population Health Science
**Primary Subject Heading**:	Ophthalmology
Secondary Subject Heading:	Ophthalmology
Keywords:	Glaucoma < OPHTHALMOLOGY, OPHTHALMOLOGY, Neuro-ophthalmology < OPHTHALMOLOGY

SCHOLARONE™ Manuscripts

I, the Submitting Author has the right to grant and does grant on behalf of all authors of the Work (as defined in the below author licence), an exclusive licence and/or a non-exclusive licence for contributions from authors who are: i) UK Crown employees; ii) where BMJ has agreed a CC-BY licence shall apply, and/or iii) in accordance with the terms applicable for US Federal Government officers or employees acting as part of their official duties; on a worldwide, perpetual, irrevocable, royalty-free basis to BMJ Publishing Group Ltd ("BMJ") its licensees and where the relevant Journal is co-owned by BMJ to the co-owners of the Journal, to publish the Work in this journal and any other BMJ products and to exploit all rights, as set out in our licence.

The Submitting Author accepts and understands that any supply made under these terms is made by BMJ to the Submitting Author unless you are acting as an employee on behalf of your employer or a postgraduate student of an affiliated institution which is paying any applicable article publishing charge ("APC") for Open Access articles. Where the Submitting Author wishes to make the Work available on an Open Access basis (and intends to pay the relevant APC), the terms of reuse of such Open Access shall be governed by a Creative Commons licence – details of these licences and which Creative Commons licence will apply to this Work are set out in our licence referred to above.

Other than as permitted in any relevant BMJ Author's Self Archiving Policies, I confirm this Work has not been accepted for publication elsewhere, is not being considered for publication elsewhere and does not duplicate material already published. I confirm all authors consent to publication of this Work and authorise the granting of this licence.

For peer review only

Standard automated perimetry using size III and size V stimuli in advanced stage glaucoma: an observational cross-sectional comparative study.

Devindra Sood MBBS, MS, FACS[1,2,3], Gabriela Czanner PhD CStat[4], Tobi Somerville MBChB, MRes[5], Ishaana Sood, BSc[6], Fiona J Rowe PhD[7]

1. Glaucoma Clinic, New Delhi, India

2. Glaucoma Service, Sadguru Netra Chikatsalya, Chitrakoot, India

3. Head of Academics, Research and Training, Sadguru Netra Chikatsalya, Chitrakoot, India

4. School of Computer Science and Mathematics, Liverpool John Moore's University, UK

5. Department of Eyes and Vision Science, University of Liverpool, UK

6. SK Glaucoma Care Foundation, New Delhi, India

7. Institute of Population Health, University of Liverpool, UK

Address for correspondence

Prof Fiona Rowe, Waterhouse Building, Block B, First Floor, University of Liverpool, 1-5 Brownlow Street, Liverpool, L69 3GL

E-mail: rowef@liverpool.ac.uk Twitter: @orthopticbrain

For peer review only

Word Count: 4380

Tables: 2

Figures: 3

Supplementary figures: 2

Short title: Size III vs V perimetry in advanced glaucoma

For peer review only - http://bmjopen.bmj.com/site/about/guidelines.xhtml

Abstract

Objectives: In this study, we sought to evaluate the extent of further visual field that could be assessed when using stimulus size V in standard automated perimetry compared to size III in advanced stage glaucoma and whether cut-off values could be determined for when to switch from size III to size V.

Design: Prospective cross-sectional study.

Setting: Single centre out-patient eye clinic in India (New Delhi).

Participants: Advanced stage glaucoma defined as stages 3-4.

Intervention: Central static perimetry with Octopus 900 G programme (size III stimulus dynamic strategy) and LVC programme (size V stimulus dynamic strategy).

Primary and secondary outcome measures: Visual field assessment for right and left eyes with both sizes III and V were undertaken within one clinic visit.

Results: We recruited 126 patients (170 eyes). Mean patient age at assessment was 55.86 years (SD 15.15). Means (SD) for size III versus size V respectively were 6.94dB (5.58) and 12.98dB (7.77) for mean sensitivity, 20.02dB (5.67) and 19.22dB (7.74) for mean deviation, 5.89dB (2.29) and 7.69dB (2.78) for standard loss variance and 3.32 minutes (1.07) and 6.40 minutes (1.43) for test duration. All except mean deviation were significantly different between size III and V tests.

Conclusion: Useful visual field information was obtained with size V stimuli which allowed continued monitoring of these patients that was not possible with size III. Increased test duration, standard loss variance and mean sensitivity were found with size V, as expected, given that more visual responses were obtained with the increased target size. A switch from size III to V may be considered when mean sensitivity reaches 10dB and/or mean deviation reaches 18dB.

Keywords

Standard automated perimetry; Size III; Size V; Glaucoma

Article summary – strengths and limitations of this study

For peer review only - http://bmjopen.bmj.com/site/about/guidelines.xhtml

- Size V stimulus in standard automated perimetry has a greater dynamic range than size III stimulus
- Visual field results of advanced stage 3-4 glaucoma meeting reliability criteria were analysed.
- Sample size calculations were met for recruitment to this study.

Declarations of interest

Funding: This research received no specific grant from any funding agency in the public, commercial or not-for-profit sectors.

Conflicts of interest: FR reports consultancy work for Haag Streit AG and use of perimeters for research from Haag Streit AG and Zeiss Meditec, companies that may be affected by the research reported in the enclosed paper. There are no potential conflicts of interest reported by the other authors.

Data availability: The dataset for this paper is not available for sharing.

Ethics approval: Institution ethical approval (Glaucoma Clinic New Delhi, India) was obtained and patients provided informed consent.

Author contributions: FR and DS conceived this study. DS and IS contributed to data collections. FR and TS contributed to data extraction. FR and GC contributed to data analysis. All authors contributed to writing this paper.

For peer review only

Introduction

Standard automated perimetry using stimulus size III is an internationally accepted visual field assessment for glaucoma [1]. Static threshold perimetry is particularly adept in aiding detection of early visual field loss in glaucoma. For patients with glaucoma, the usual choice of visual field assessment is static perimetry using a threshold strategy to assess the central 24 to 30 degrees of the visual field of right and left eyes individually [1]. In clinical practice a number of issues can arise with these central assessment programmes. As glaucoma progresses and the central

For peer review only - http://bmjopen.bmj.com/site/about/guidelines.xhtml

visual field deteriorates to the extent that minimal (if any) visual field remains, appropriate and reliable evaluation can become difficult.

For patients with severe to advanced stage disease, central visual field assessment can show substantial visual field loss and, therefore, is limited in providing useful information about further progression or stability of the visual field loss, or for providing information about the individual's functional visual status. In these patients, further analysis options for visual field assessment must be considered. Where the central visual field island remains, it is possible to target assessment to the central 10 degrees of visual field. Tomairek and colleagues [2] showed a considerable number of eyes with severe glaucoma with absolute visual field defects on 24-2 programme testing showing relative defects involving only some points of the central 10 degrees on the 10-2 programme. Thus, they stressed the importance of detailed central testing to underpin intensive treatment to preserve the residual central visual field.

A further alternative is to continue assessment of the central 30 degrees but to increase the stimulus to size V [3-5]. Whilst there is considerable debate over the use of different stimulus sizes in automated perimetry [6-10], from a clinical perspective, size III remains the standard stimulus in mainstream clinical use [1,7]. However, when visual field results using standard automated perimetry show severe loss, use of size V stimulus becomes a consideration.

There are conflicting reports of size V versus III in early visual field loss. The use of the larger stimulus size could prevent the detection of small localised defects because the large stimulus size stimulates not only the area of visual field loss but also the surrounding, better, areas of visual field [6]. However, Wall and colleagues [5] found that when the data was binned by mean deviation, size V maintained its advantage to detect visual loss even in the bin with the best mean deviation. Further, Flanagan and colleagues [11] reported that while size III testing resulted in a greater depth of the defect, size V, because of its better retest variability, flagged abnormal test locations in early glaucoma at least as well as size III. This is not directly relevant to studies that focus specifically on severe visual field loss. With severe visual field loss, when using size III, it is not possible to gather reliable visual field data by increasing stimulus contrast (i.e. lower decibel values) as often there is no

For peer review only

response within damaged areas of visual field [6,7]. Thus, a further option is to increase the target size instead.

The useful dynamic range of perimetry is the range of disease severities over which useful measurements can be obtained. With increasing visual field loss, the response within the area of visual field loss becomes more variable because of decreased retinal ganglion cell response rate along with the limited number of stimulus presentations in each location of the test programme [6,7]. Use of size V stimulus has been shown to have a greater dynamic range and a similar number of abnormal test locations when compared to the traditionally used size III stimulus [3]. Higher sensitivity is achieved at the same location giving more reliable and less variable estimates of sensitivity at the damaged visual field locations. Gardiner reported that for rapidly progressing eyes, use of size V could represent more than 5 years of added, useful and reliable test results. For less rapid loss, size V could provide many years of reliable clinical information [6].

In this study we aimed, in a pragmatic clinical study, to investigate the information obtained from central static visual field assessment comparing size V versus size III targets using the Octopus 900 G and LVC programmes in patients with advanced stage glaucoma. We hypothesise that, for many patients, we would obtain useful visual field results with size V to use as a new baseline for visual field status for subsequent comparison over follow-up, and further, comparative data of size III and V could be used to provide a recommendation for transfer from one test strategy to the other.

Materials and methods

Design

We undertook a prospective, comparative, observational cross-sectional study in a single centre in New Delhi, India. Institution ethical approval (Ethics committee, Glaucoma Clinic New Delhi, India: Registration number GCND/EC/108) was obtained and patients provided informed consent. We followed the STROBE checklist for reporting cross-sectional studies.

For peer review only

Recruitment

The study recruited patients with visual field loss due to advanced glaucoma and who met our inclusion criteria. Consecutive patients with an existent clinical diagnosis of glaucoma requiring visual fields were recruited from ophthalmology outpatient clinics between 2016-2017. Severe glaucoma was diagnosed according to the International Classification of Diseases (ICD) diagnostic code 9 (365.73) and 10 (7th digit “3”). Diagnostic code 365.73 represents the glaucoma stage code for severe, advanced and end-stage glaucoma consisting of “glaucomatous visual field abnormalities in both hemifields and/or loss within 5 degrees of fixation in at least one hemifield” [12,13]. ICD version 9 codes were updated in 2015 to version 10 codes which consist of seven digits [14]. The first three indicate the code category and the last four provide added detail. Where the last digit is “3”, this indicates severe glaucoma regardless of the type or cause of glaucoma. For example, H4010X3 represents unspecified open angle severe stage glaucoma.

Inclusion criteria

We included adult patients attending for visual field assessment with a diagnosis of severe or advanced glaucoma (stages 3-4), sufficient motor ability to sit at the perimeter unaided, ability to press the response button, sufficient cognitive ability to understand and follow instructions for performing the test, and willingness to undergo standard visual field assessments on the same day.

Exclusion criteria

We excluded patients with a diagnosis of stage 1-2 glaucoma, visual acuity worse than 1.0 logMAR, those unable to sit at the perimeter, those with unreliable visual fields, unable to follow instructions for performing the test or too ill to complete the full assessment.

Patient and public involvement

This study addresses a top research priority identified by patients and the public in a national (UK) consultation process: “what is the most effective way of monitoring the progression of glaucoma?” (https://www.jla.nihr.ac.uk/). Patients were not involved directly in the design and conception of this study.

For peer review only

Visual field assessment

Past studies have frequently used the 24-2 programme when testing visual fields in advanced glaucoma [3-5]. The Octopus 900 perimeter (Haag Streit AG, Bern, Switzerland) G programme (dynamic strategy) has a physiology-based grid of 59 test locations within the central 30 degrees [15]. Locations are clustered more closely together centrally (2.8 degree spacing) with five central foveal locations and 17 test locations in the macular region. Locations are spaced further apart peripherally with emphasis on locations in nasal step regions and with more test locations nasally than temporally. There is no weighted analysis of test locations. Test locations are distributed in a pattern to follow retinal nerve fibre bundles to facilitate the detection of glaucomatous visual field loss. The Octopus 900 perimeter low vision central (LVC) programme (dynamic strategy) has a grid of 75 test locations within the central 30 degrees [15]. One location is within the central fixation area and the remaining 74 locations are spaced equally apart by 6 degrees with an off-set from the horizontal and vertical meridians of 2 degrees. Static visual field results were deemed unreliable if combined false negative and false positive responses exceeded 25%.

The study protocol consisted of visual field assessment with static G Octopus 900 perimetry using size III and static LVC size V targets (two tests per eye) on the same day. The order of assessment was randomised for size III versus size V. The G and LVC programmes were run as standard with the size III target size (0.43 degrees in diameter) or with the size V target size (1.72 degrees in diameter).

Sample size

We studied two main outcomes: mean sensitivity and mean deviation.

For mean sensitivity sample size we assumed a difference of 3dB to be of clinical importance. Wall et al. [5] reported lower and upper quartiles of 17.4dB and 24.5dB which are equivalent to a standard deviation of 3.55. Based on these figures, with pairwise t-test, significance level of 2.5% and power of 85%, this leads to a sample size of 15 eyes (one eye per patient).

For mean deviation, in the sample size we assume a difference of 10% decrease to be clinically relevant. Wall et al [4] showed a 15% decrease in their patients, while pooled standard deviation was 5.5dB7. With pairwise t-test, and significance level of

For peer review only - http://bmjopen.bmj.com/site/about/guidelines.xhtml

2.5%, and power of 85%, this leads to a sample size of 126 eyes (one eye per patient).

Statistical analysis

A direct comparison of results was made for static results using the statistical package SPSS version 25 (IBM SPSS Statistics, USA). The unit of analysis was per eye [16].

Glaucoma typically affects both eyes, thus each eye is not considered independent when considering analysis of visual field assessments. Assessments are usually reported for worst affected eye only. In this study we sought to evaluate visual field data available from size V versus size III perimetry. We had all the data from both eyes for many patients, hence we aimed to utilise all the data from all eyes as our primary analysis. For the purposes of sensitivity analysis, we also present results from worst eyes only [16]. We chose to include worst eyes only for this analysis on the basis that we wished to determine at what point within worst fields we should transfer from size III to size V testing. Worst eye was defined as the eye with greater mean deviation value on G1 static perimetry.

To evaluate normality of distribution of results from right and left eyes, a Kolmogorov-Smirnov test was used. Further we conducted a general linear model analysis for difference between eyes in mean sensitivity and mean deviation. For this model, the difference in mean sensitivity, or mean deviation, value was the dependent variable and the eye (right versus left nested within the patient) was treated as random factor. We also did analysis of the visual field measurement difference in the worse eye via a paired t-test with Bonferroni adjustment for mean sensitivity and mean deviation calculations.

To compare the results of the G programme when undertaken with size III and size V targets, variation was evaluated for:

- Mean sensitivity
- Mean deviation
- Standard loss variance

For peer review only - http://bmjopen.bmj.com/site/about/guidelines.xhtml

We further compared mean sensitivity and mean deviation values for each of the four quadrants of the visual field to determine if different quadrants showed comparable changes. Quadrants were defined as Q1 (superior nasal field), Q2 inferior nasal field), Q3 (superior temporal field) and Q4 (inferior temporal field) for both right and left visual field results. Duration of assessments was also compared.

Scatterplots include linear regression lines and Loess curves. The linear regression line assesses strength of linear association between two continuous variables with r^2 =0.0 indicating lack of linear association (i.e. lack of correlation). Correlation is first assessed by its p-value (p) and then, if significant, the correlation is judged as none, small, medium or large if r^2 is 0-0.01, 0.01-0.09, 0.09-0.25 or more than 0.25 [17,18]. A non-directional p-value, i.e. two-sided alternative hypothesis, is used. The value of r^2 tells us how much variability is shared between variables. The Loess (locally estimated scatterplot smoothing) curve is a local nonlinear regression curve in which the fitting of each point is weighted towards the data nearest to that point. It makes no assumption about the association between variables and is used to visually observe the possible nature of the association between two continuous variables. It can be used as a modelling tool and provides a nonparametric regression that focuses on the fitted curve [19]. The fitted points and related standard errors do respect a particular estimate but are estimated to the whole curve [20]. The default span was set to values ranging from 0.60-0.80 as a trade-off to ensure sufficient data for an accurate fit in order to reduce variance, and to avoid an over-smoothed regression in order to reduce bias. Along the Loess curve, the cross section between the x and y axis relating to the main point of inflection along the curve was used to indicate cut-off values between data represented along the x and y axes and therefore, for this study, used to calculate potential cut-off values for transfer of testing from size III to size V stimulus. The inflection point was taken to indicate at what value the relationship between III and V was possibly changing (as relevant when the linear relationship does not explain the association between III and V). The point of greatest curvature is suggested as a potential point of transitioning from size III to V as that is the point when the size III stimulus accelerates in its inability to detect change in visual field – relatively when compared to size V test. We report the inflection point as a range of values, as observed for the range of (α) 0.60-0.80.

For peer review only - http://bmjopen.bmj.com/site/about/guidelines.xhtml

In this paper all global indices are reported as positive numerical values, including mean sensitivity, mean deviation and standard loss variance.

Results

Primary analysis: All patients / eyes

Results are presented for 126 patients (170 eyes) with reliable visual field performance; 29 eyes at stage 3 and 141 at stage 4. A further 46 eyes were excluded because of unreliable visual field performance or visual field results graded at stage 2 or better. The worst affected eye was right in 64 (50.8%) and left in 62 (49.2%). Mean age at time of testing was 55.86 years (SD 15.15; 58 [range 13-83]). Figure 1 displays one example of size III versus size V results for one patient.

Distribution of data

Visual field assessment data was tested to be normally distributed (Kolmogorov-Smirnov test). Further, general linear model analysis with random effect being the subject and fixed effect being 1) eye laterality (right versus left eye) or 2) worst

For peer review only - http://bmjopen.bmj.com/site/about/guidelines.xhtml

Table 1: Differences in visual field parameters for target size – all eyes

	G1, size III		G1, size V		Difference				
	Mean	SD	Mean	SD	Mean	SD	General linear mixed model (subject)	Correlation significance	Bonferroni correction
MS	6.94	5.58	12.98	7.77	-6.04	4.18	0.0001	0.005	0.0002
MD	20.04	5.67	19.22	7.74	0.82	4.15	0.023	0.007	0.046
SLV	5.89	2.29	7.69	2.78	-1.80	2.60	0.0001	0.0001	*
DD	17.13	7.86	14.76	9.47	2.37	5.46	0.0001	0.009	*
MS Q1	5.31	5.48	10.48	8.78	-5.17	5.21	0.0001	0.0001	*
MS Q2	7.70	7.48	14.40	9.74	-6.70	5.33	0.0001	0.004	*
MS Q3	6.27	5.78	12.05	8.68	-5.77	5.42	0.0001	0.114	*
MSQ4	8.23	7.59	14.68	10.12	-6.45	5.37	0.0001	0.005	*
MD Q1	21.29	5.55	21.13	8.70	0.16	5.16	0.662	0.001	*
MD Q2	19.59	7.58	18.28	9.77	1.31	5.31	0.010	0.005	*

For peer review only

For peer review only - http://bmjopen.bmj.com/site/about/guidelines.xhtml

MD Q3	20.25	5.86	19.58	8.60	0.67	5.37	0.119	0.123	*
MD Q4	19.06	7.61	18.00	10.08	1.06	5.37	0.018	0.006	*
Duration	3.32	1.07	6.40	1.43	-3.07	1.66	0.0001	0.015	*

MS – mean sensitivity; MD – mean deviation; SLV – standard loss variance; DD – diffuse defect; Q1 (superior nasal field); Q2 inferior nasal field); Q3 (superior temporal field); Q4 (inferior temporal field).

* Not corrected as study not powered for these comparisons

For peer review only

Table 2: Differences in visual field parameters for target size – worst eyes

	**G1, size III**		**G1, size V**		**Difference**			
	Mean	SD	Mean	SD	Mean	SD	Paired t test	Bonferroni correction
**MS**	6.31	5.57	12.24	7.67	-5.93	4.43	0.0001	0.0002
**MD**	20.72	5.63	20.00	7.59	0.72	4.39	0.072	0.144

**SLV**	5.73	2.34	7.67	2.70	-1.94	2.59	0.0001	*
**DD**	18.13	7.84	15.58	9.44	2.54	5.78	0.0001	*
**MS Q1**	4.80	5.38	9.92	8.57	-5.11	5.47	0.0001	*
**MS Q2**	7.00	7.52	13.64	9.61	-6.64	5.36	0.0001	*
**MS Q3**	5.73	5.85	11.26	8.74	-5.52	5.80	0.0001	*
**MS Q4**	7.49	7.44	13.86	10.06	-6.37	5.65	0.0001	*
**MD Q1**	21.88	5.42	21.75	8.46	0.12	5.42	0.801	*
**MD Q2**	20.34	7.57	19.08	9.60	1.27	5.34	0.01	*
**MD Q3**	20.85	5.93	20.42	8.62	0.43	5.73	0.407	*
**MD Q4**	19.85	7.46	18.86	9.99	0.99	5.64	0.053	*
**Duration**	3.31	1.07	6.36	1.37	-3.05	1.65	0.0001	*

MS – mean sensitivity; MD – mean deviation; SLV – standard loss variance; DD – diffuse defect; Q1 (superior nasal field); Q2 inferior nasal field); Q3 (superior temporal field); Q4 (inferior temporal field).

* Not corrected as study not powered for these comparisons

For peer review only - http://bmjopen.bmj.com/site/about/guidelines.xhtml

versus better eye, with respect to mean sensitivity, showed no significant difference between size V versus size III data (p=0.888 and p=0.644 respectively).

Size III versus size V differences

Results are outlined in table 1 for differences, and significance, for mean sensitivity, mean deviation and standard loss variance, along with differences across quadrants plus test duration. Differences for mean sensitivity between sizes III and V were about 6 decibels overall (higher values for size V) and 5-7 decibels across quadrants with greatest increases in inferior quadrants. A strong linear relationship and large correlation was found between mean sensitivity for size III and V stimuli (Figure 2: r^2=0.731, p=0.0001). Using the Loess curve to consider differences between size III and V stimuli, the point of greatest inflection (visualised on the scatterplot with value taken from y axis) is approximately 4-6 decibels for size III stimulus.

Global mean deviation was significantly lower for size V but not clinically significant with significant differences only in inferior quadrants. A strong linear relationship and large correlation was found between mean deviation for size III and V stimuli (Figure 2: r^2=0.735, p=0.0001). Using the Loess curve to consider differences between size III and V stimuli, the point of greatest inflection is approximately 22-24 decibels for size III stimulus. Diffuse defect was lower and standard loss variance was higher for size V (see Supplementary Figure 1) representing more visual field responses with the size V target. Test duration increased for size V but was likely due to more patient responses being obtained due to presence of more visible targets.

Sensitivity analysis: Worst eyes only

Taking only the worst eye for each patient, results are presented for 126 patients (126 eyes) with 18 eyes at stage 3 and 108 at stage 4. The worst eye was right in 64 (50.8%) and left in 62 (49.2%).

Size III versus size V differences

Results are outlined in table 2 for differences, and significance, for mean sensitivity, mean deviation and standard loss variance, along with differences across quadrants plus test duration. Differences for mean sensitivity between size III and V were about 6 decibels overall (higher values for size V) and 5-7 decibels across quadrants with

For peer review only - http://bmjopen.bmj.com/site/about/guidelines.xhtml

greatest increases in inferior quadrants. A strong linear relationship and large correlation was found between mean sensitivity for size III and V stimuli (Figure 3: r^2=0.678, p=0.0001). Using the Loess curve to consider differences between size III and V stimuli, the point of greatest inflection (visualised on the scatterplot with value taken from y axis) is approximately 3-5 decibels for size III stimulus.

Global mean deviation was significantly lower for size V but not clinically significant (Figure 3) with significant differences only in inferior quadrants. A strong linear relationship and large correlation was found between mean deviation for size III and V stimuli (Figure 3: r^2=0.687, p=0.0001). Using the Loess curve to consider differences between size III and V stimuli, the point of greatest inflection is approximately 22-24 decibels for size III stimulus. Diffuse defect was lower and standard loss variance was higher for size V (see Supplementary Figure 2) representing more visual field responses. Test duration increased for size V but was likely due to more patient responses being obtained due to presence of more visible targets.

Discussion

Detection of visual field loss in glaucoma and other optic neuropathies is well reported with discussion of early detection methods, exploring visual field defects with steep border slopes and for conversion of disease/progression [1,21]. Studies have mostly used the 24-2 central static threshold programme [3-5]. In this study we sought to explore the use of size V versus size III targets using the Octopus 900 G and LVC programmes to evaluate detection of absolute versus relative visual field loss in advanced (stages 3-4) glaucoma. This was a pragmatic clinical study in which we primarily wished to evaluate how much additional useful information could be obtained from a switch to stimulus size V at a stage of moderate to severe glaucoma when visual field results had reached a level of loss with stimulus size III, such that meaningful information could no longer be extracted from the results to guide clinical decision making.

One recent study sought to gather patient perspectives and reliability based on clinical grounds whilst comparing size III and V stimulus sizes using a 24-2 test

For peer review only - http://bmjopen.bmj.com/site/about/guidelines.xhtml

For peer review only

programme [22]. They concluded that adjustment of testing to use of size V increased their patient cooperation and was a valid option when attempting to gather more reliable perimetry results. From our clinical perspective, we found the results using size V to provide more visual field data to help inform clinical decision making and a grounding towards future disease monitoring.

The mean sensitivity provides a single global indicator value for the overall visual field sensitivity. We found the mean sensitivity value to increase overall by about 6 decibels for size V versus size III but with a difference of about 10 decibels (in keeping with that expected by the change in stimulus size) at mean sensitivities of 10-30 decibels. This indicates less visual field loss for size V stimulus compared to the indication of absolute defects for size III stimulus when mean sensitivity values are low (<10 decibels) and is indicative of the relationship between stimulus size and luminance [22]. Many perimeter parameters are such that at maximum luminance, the size III target has a calibrated range of 2-44 decibels and the size V target has a range of 10-54 decibels (10 decibel difference). As expected, the larger stimulus size increases sensitivity and this is particularly so at greater eccentricities [6]. In clinical studies this is shown as the effective dynamic range of size V being about one log unit greater than size III [3]. Gardiner and colleagues confirmed size V resulted in higher sensitivity at the same location, providing more reliable and less variable estimates of sensitivity in damaged visual fields. Their higher sensitivity was on average 5.6 decibels – similar to our average of 6 decibels and to the average of 6 and 7.6 decibels reported by Morgan et al [22] and Choplin et al [8], respectively.

The mean deviation provides a single global indicator value for the overall visual field sensitivity in comparison to an age-matched normal visual field. We found a slight but clinically insignificant reduction in mean deviation for size V versus III. The association between size III and V targets has been reported as approximately linear to a sensitivity of about 20 decibels. Size V had a greater effective dynamic range with eight discriminable steps for progression – about twice as many as with size III [23].

A second objective of our study was to consider indication levels for a transfer from size III to size V for static perimetry. In our analysis, we used the Loess curve to compare differences between size III and V stimuli. The Loess curve does not

For peer review only

For peer review only - http://bmjopen.bmj.com/site/about/guidelines.xhtml

provide confidence values. We found the point of inflection to be within an overall range of approximately 4-6 decibels for size III stimulus on mean sensitivity and 22-24 decibels on mean deviation. Arguably, the inflection point might be considered the latest point at which to switch from size II to size V. A more sensible approach would be to switch before severe damage to the visual field occurs to allow progression to be more reliably tracked with size V over, potentially, a longer period of time. We therefore provide a clinical recommendation that at a mean sensitivity level of 10 decibels (4 decibels above the higher inflection estimate) and/or at a mean deviation level of 18 decibels (4 decibels below the lower inflection estimate) on a visual field using size III, a switch may be made to using size V stimuli for subsequent visual field assessment.

Mean deviation reduced more for inferior visual field quadrants. Our discrepancy between superior and inferior visual field cannot be explained by the number of test locations as test location numbers are equal for the superior and inferior visual field. The greater reduction in mean deviation for inferior visual field may be explained by the presence of better inferior visual field compared to the extent of superior visual field loss. In both primary open and closed angle glaucoma, visual field damage has often been reported as being more severe in the superior visual field than inferiorly [24].

Variability for size V targets has been reported as being substantially lower than for size III [3]. However, in later studies, Wall and colleagues [4,5] did not find a significant reduction in size V variability compared to size III. This was partly explained by the use of size V allowing patients to detect more targets than with the smaller size III – 0 decibel responses using size III become detectable decibel responses with size V. Thus, as patients can see more, the extra responses add to variability. This was not apparent in cases with most severe visual field loss with mean sensitivities close to 0 decibel but emerged from a mean sensitivity of 10 decibels or higher, i.e. when sufficient visual field was discernible. We found mean deviation to be higher for size III generally up to 20-25 decibels at which level mean deviation for size V was higher which likely reflects the greater extent of visual field seen with size V than size III. This effect disappeared for very poor visual fields, even with size V. Our study only included stage 3-4 advanced glaucoma with cases where there was substantial visual field loss to the extent that perimetry with size III

For peer review only - http://bmjopen.bmj.com/site/about/guidelines.xhtml

For peer review only

no longer provided adequate information to facilitate monitoring of the condition for progression/response to treatment. Changing to size V allowed us to further evaluate visual fields with more visual field information being obtained.

The standard loss variance provides a single global indicator value for the extent of localised visual field loss that is present. Our results indicate higher standard loss variance measures with size V likely due to greater visual field responses being obtained with the larger size target. Further, and not surprising, we found longer test durations for size V compared to size III and in keeping with other studies [22]. This reflects the great visual response to the larger size V target. Thus, the more targets than can be detected and responded to, the longer the test time will be.

We addressed a methodological issue with this paper. Many studies reporting visual field analysis present data from one eye: often the worst eye. We evaluated the distribution of data for all eyes in this study as our primary analysis. We subsequently ran a sensitivity analysis of worst eyes only to determine where significance lies when only focussing on the worst eye and where the sample size is smaller. We found no differences in distribution for right versus left eyes or for worst versus better eyes except for mean deviation which showed more significance for primary analysis and which likely reflects the larger sample size for primary analysis (all eyes). Such evaluation of distribution is essential if wishing to combine results from all eyes in reporting data. Clearly analysis and reporting of data is important in the context of the type of study. Reporting intervention outcomes often necessitates a determination of independent effect. Hence reporting data from one eye per subject is often appropriate where the condition affects both eyes. However, in studies of assessment comparisons (such as this present study) the question being asked is about the ability of each assessment/test to detect the same defect and that question is as important for each eye individually (as two independent comparisons) as for the patients individually (as a single comparison). We have reported our methods and results in establishing the distribution of data and our subsequent decision to combine results versus solely presenting worst eye data only. As seen from our results across tables 1 and 2, values were highly comparable for all eyes versus worst eyes only.

Limitations

For peer review only - http://bmjopen.bmj.com/site/about/guidelines.xhtml

Our study is limited by a number of factors. We report visual field results but did not collect visual acuity or fundus imaging for this study. We also did not formally record patient preference for visual field testing with size III or V. Further, we did not evaluate test reliability by accounting for false positive and false negative responses, although excessive responses were exclusion criteria for our study. We do not believe these are major limiting factors as we considered this a pragmatic clinical study centred on evaluating the differing visual field results obtained from size III and V stimuli. Hence, visual acuity and fundus imaging would not impact on our analysis. In future studies, we would wish to include patient feedback on preference for stimulus size as better patient engagement with visual field assessment is important.

Conclusions

Use of size V versus III in standard automated perimetry for advanced stages 3-4 in glaucoma permits further visual field to be plotted in cases where the visual fields with size III no longer provides sufficient information to appropriately monitor the disease. Mean sensitivity, standard loss variance and test duration increased in accordance with the extra visual field responses obtained using size V. We do not infer that automated perimetry with size V is better than with size III. Size V is easier to detect and, thus, from a pragmatic clinical view, a switch from size III to V in patients with advanced disease was a logical alteration to the visual field assessment protocol. We recommend considering a change to size V stimulus for advanced glaucoma at stages 3-4 when, with size III, the mean sensitivity reaches 10 decibels and/or mean deviation reaches 18 decibels, to facilitate continued assessment of visual fields as part of the clinical monitoring of the condition.

References

For peer review only - http://bmjopen.bmj.com/site/about/guidelines.xhtml

1. Bua S, Supuran CT. (2019) Diagnostic markers for glaucoma: a patent and literature review (2013-2019). Expert Opin Ther Pat. doi: 10.1080/13543776.2019.1667336
2. Tomairek RH, Aboud SA, Hassan M, Mohamed AH. (2019) Studying the role of 10-2 visual field test in different stages of glaucoma. Eur J Ophthalmol. 1-8: DOI: 10.1177/1120672119836904
3. Wall M, Brito CF, Woodward KR, Doyle CK, Kardon RH, Johnson CA. (2008) Total deviation probability plots for stimulus size V perimetry. Arch Ophthalmol. 126: 473-479
4. Wall M, Doyle CK, Zamba KD, Artes P, Johnson CA. (2013) The repeatability of mean defect with size III and size V standard automated perimetry. Invest Ophthalmol Vis Sci. 54: 1345-1351
5. Wall M, Doyle CK, Eden T, Zamba KD, Johnson CA. (2013) Size threshold perimetry performs as well as conventional automated perimetry with stimulus sizes III, V and VI for glaucomatous loss. Invest Ophthalmol Vis Sci. 54: 3975-3983
6. Gardiner SK, Demirel S, Goren D, Mansberger SL, Swanson WH. (2015) The effect of stimulus size on the reliable stimulus range of perimetry. Tran Vis Sci Tech. 4(2): 10, http://tvstjournal.org/doi/full/10. doi:10.1167/tvst.4.2.10
7. Swanson WH. (2013) Stimulus size for perimetry in patients with glaucoma. Research highlight. Invest Ophthalmol Vis Sci. 54(6):3984
8. Choplin NT, Sherwood MB, Spaeth GL. (1990) The effect of stimulus size on the measured threshold values in automated perimetry. Ophthalmol. 97:371–374.
9. Gilpin L, Stewart W, Hunt H, Broom C. (1990) Threshold variability using different Goldmann stimulus sizes. Acta Ophthalmol. 68:674–676.
10. Wall M, Kutzko K, Chauhan B. (1997) Variability in patients with glaucomatous visual field damage is reduced using size V stimuli. Invest Ophthalmol Vis Sci. 38:426–435.
11. Flanagan JG, Artes PH, Wall M, Young E, Callan T, Patella M, Monhart M, Lee GC. Influence of Perimetric Stimulus Size on Defect Detectability in Early Glaucoma. Invest Ophthalmol Vis Sci 2016 (abstract); 58(8):2842. https://iovs.arvojournals.org/article.aspx?articleid=2557916

For peer review only - http://bmjopen.bmj.com/site/about/guidelines.xhtml

12. WuDunn D. (2015) Monitoring patients with advanced glaucoma. Glaucoma Today. September/October: 42-43
13. Fellman RL, Mattox CG, Ross KM, Vicchrilli S. (2011) Know the new glaucoma staging codes. Eyenet. October: 65-66
14. https://icd.who.int/browse10/2010/en#/H40-H42 [last accessed 12th May 2020]
15. Racette L, Fischer M, Bebie H, Hollo G, Johnson CA, Matsumoto C. (2016) Visual field digest. A guide to perimetry and the Octopus perimeter. 6th edition. Haag Streit AG, Switzerland
16. Bunce C, Patel KV, Xing W, Freemantle N, Dore CJ, (2014) Ophthalmic Statistics Group. Ophthalmic statistics note 1: unit of analysis. Br J Ophthalmol. 98: 408-12
17. Illowsky B, Dean S. (2013) Introductory Statistics. OpenStax, Houston, Texas. https://openstax.org/books/introductory-statistics/pages/12-4-testing-the-significance-of-the-correlation-coefficient [accessed 29th May 2020]
18. Cohen J. (1988) Statistical power analysis for the behavioural sciences. Routledge. ISBN 978-1-134-74270-7
19. Cleveland WS, Grosse E, Shyu WM. (1992) Local regression models. Chapter 8. Statistical Models in S. Editors: Chambers JM and Hastie TJ, Wadsworth & Brooks/Cole
20. https://www.statsdirect.com/help/nonparametric_methods/loess.htm [accessed 29th May 2020]
21. Peters D, Bengtsson B, Heijl A. (2015) Threat to fixation at diagnosis and lifetime risk of visual impairment in open-angle glaucoma. Ophthalmol. 122:1034-9
22. Morgan AM, Mazzoli LS, Caixeta-Umbelino C, Kasahara N. (2019). Expediency of the Automated Perimetry Using the Goldmann V Stimulus Size in Visually Impaired Patients with Glaucoma. Ophthalmol Ther, 8(2), 305–311
23. Wall M, Woodward KR, Doyle CK, Zamba G. (2010) The effective dynamic ranges of standard automated perimetry sizes III and V and motion and matrix perimetry. Arch Ophthalmol. 128: 570-576
24. Yousefi S, Sakai H, Murata H, Fujino Y, Garway-Heath D, Weinreb R, Asaoka R. (2018) Asymmetric Patterns of Visual Field Defect in Primary Open-Angle and Primary Angle-Closure Glaucoma. Invest Ophthalmol Vis Sci. 59:1279-1287

For peer review only - http://bmjopen.bmj.com/site/about/guidelines.xhtml

Figure legends

Figure 1: Illustrative results of static size III versus size V perimetry

A – G1 static perimetry with size III, left eye

B – LVC static perimetry with size V, left eye

Static perimetry (using size III) results shows a severely restricted visual field whilst static perimetry (using size V) results display better temporal visual field and inferonasal visual field.

Figure 2: Mean sensitivity and mean deviation comparisons – Primary analysis of all eyes.

A – Mean sensitivity

Straight line is the linear regression line; curved line is the Loess regression curve.

Comparisons generally show clustering where mean sensitivity is close to 0 decibels, i.e. worst visual field. There is a “floor” effect with size III, yielding many zeros, while size V gives, for most, larger values.

The linear regression line and Loess regression curve show large correlation for size V (r^2=0.731, p<0.0001). The Loess point of inflection (value taken from the y axis) is approximately 4-6 decibels.

B – Mean deviation

Straight line is the linear regression line; curved line is the Loess regression curve.

The linear regression line and Loess regression curve show large correlation for size V (r^2=0.735, p<0.0001). The Loess point of inflection (value taken from the y axis) is approximately 22-24 decibels.

Figure 3: Mean sensitivity and mean deviation comparisons – Sensitivity analysis of worst eyes.

A – Mean sensitivity

Straight line is the linear regression line; curved line is the Loess regression curve.

Comparisons generally show clustering where mean sensitivity is close to 0 decibels, i.e. worst visual field. There is a “floor” effect with size III, yielding many zeros, while size V gives, for most, larger values.

For peer review only - http://bmjopen.bmj.com/site/about/guidelines.xhtml

The linear regression line and Loess regression curve show large correlation for size V (r^2=0.678, p<0.0001). The Loess point of inflection (value taken from the y axis) is approximately 3-5 decibels.

B – Mean deviation

Straight line is the linear regression line; curved line is the Loess regression curve.

The linear regression line and Loess regression curve show large correlation for size V (r^2=0.687, p<0.0001). The Loess point of inflection (value taken from the y axis) is approximately 22-24 decibels.

Supplementary Figure 1: Standard loss variance, diffuse defect and test duration comparisons – Primary analysis of all eyes.

A: Standard loss variance

B: Diffuse defect

C: Test duration

Straight line is the linear regression line; curved line is the Loess regression curve. Loss variance is variable for sizes III and V but with higher loss variance for size V at 8-12 decibels. The linear regression line and Loess regression curve show medium to large correlation for size V (r^2=0.257, p<0.0001). The Loess point of inflection (value taken from the y axis) is approximately 5-7 decibels (1a).

Diffuse defect is higher for size III at 10-20 decibels but higher for size V at 25-30 decibels. The linear regression line and Loess regression curve show large correlation for size V (r^2=0.672, p<0.0001). The Loess point of inflection (value taken from the y axis) is approximately 23-25 decibels (1b).

Test duration increased for size V versus size III. The linear regression line and Loess regression curve show small correlation for size V (r^2=0.040, p<0.009). The Loess point of inflection (value taken from the y axis) is approximately 3-5 minutes (1c).

Supplementary Figure 2: Standard loss variance, diffuse defect and test duration comparisons – Sensitivity analysis of worst eyes.

A: Standard loss variance

For peer review only - http://bmjopen.bmj.com/site/about/guidelines.xhtml

B: Diffuse defect

C: Test duration

Straight line is the linear regression line; curved line is the Loess regression curve. Loss variance is variable for sizes III and V but with higher loss variance for size V at 8-12 decibels. The linear regression line and Loess regression curve show medium to large correlation for size V ($r^2=0.260$, $p<0.0001$). The Loess point of inflection (value taken from the y axis) is approximately 4-6 decibels (2a).

Diffuse defect is higher for size III at 10-20 decibels but higher for size V at 25-30 decibels. The linear regression line and Loess regression curve show large correlation for size V ($r^2=0.635$, $p<0.0001$). The Loess point of inflection (value taken from the y axis) is approximately 21-23 decibels (2b).

Test duration increased for size V versus size III. The linear regression line and Loess regression curve show small correlation for size V ($r^2=0.036$, $p<0.035$). The Loess point of inflection (value taken from the y axis) is approximately 3-5 minutes (2c).

For peer review only

For peer review only - http://bmjopen.bmj.com/site/about/guidelines.xhtml

1A – G1 static perimetry with size III, left eye

1B – G1 static perimetry with size V, left eye

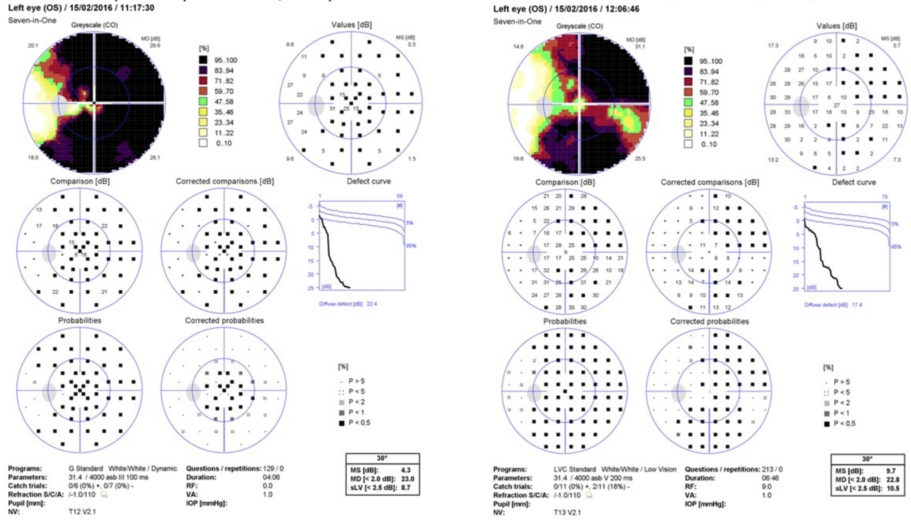

338x190mm (96 x 96 DPI)

2A – Mean sensitivity

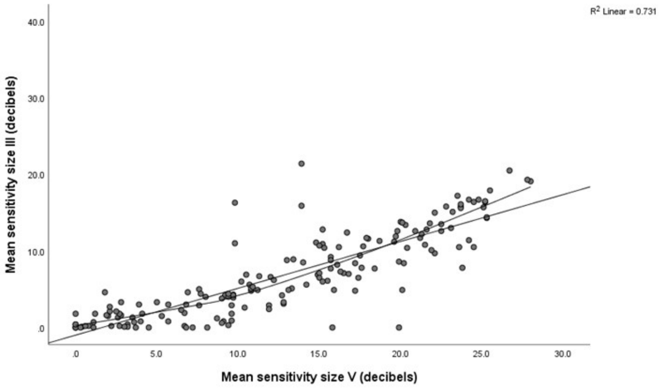

2B – Mean deviation

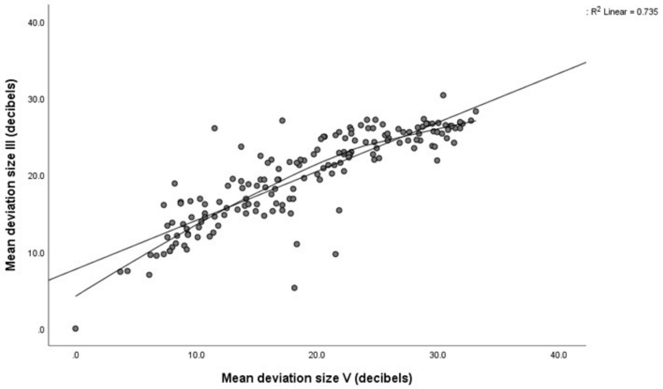

190x338mm (96 x 96 DPI)

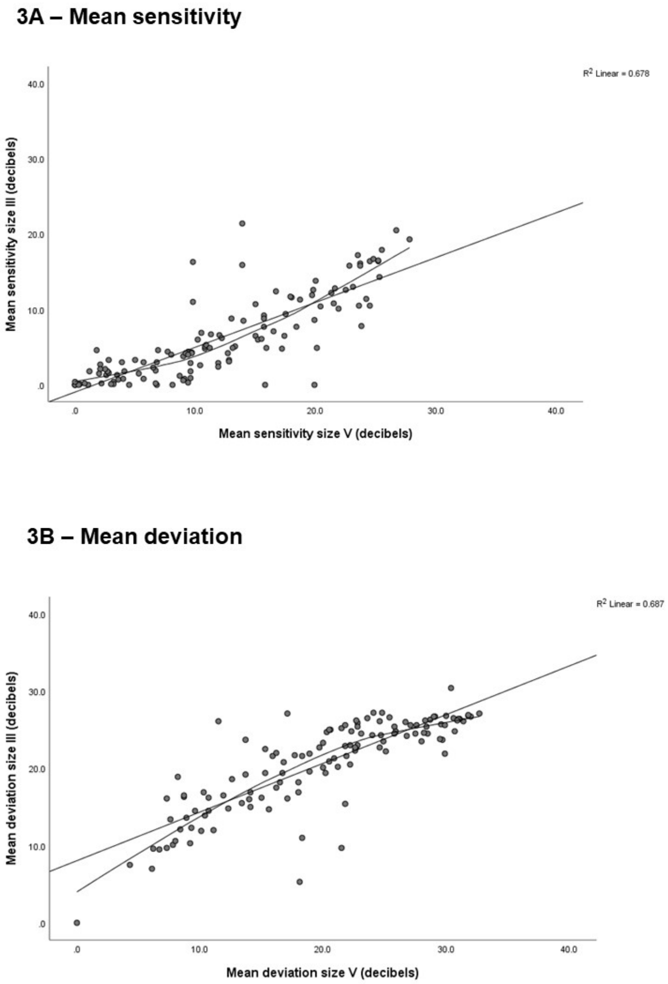

190x338mm (96 x 96 DPI)

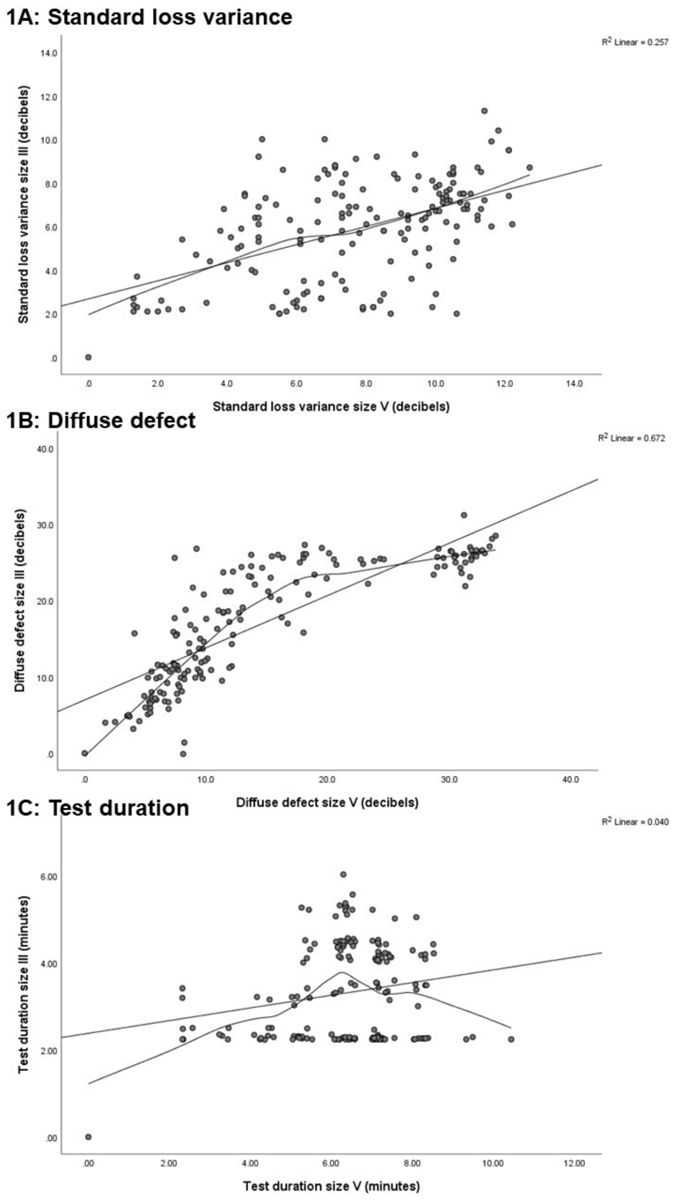

190x338mm (96 x 96 DPI)

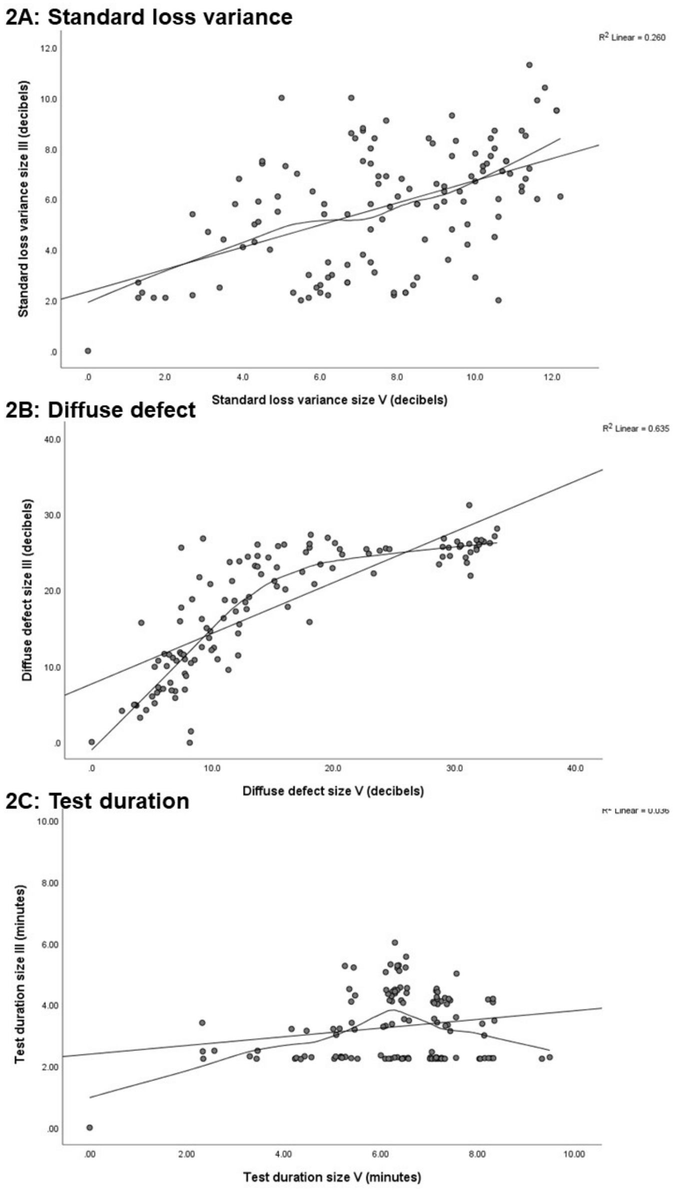

190x338mm (96 x 96 DPI)

STROBE Statement—Checklist of items that should be included in reports of ***cross-sectional studies***

	Item No	Recommendation	Page No
**Title and abstract**	1	(*a*) Indicate the study's design with a commonly used term in the title or the abstract	1
		(*b*) Provide in the abstract an informative and balanced summary of what was done and what was found	2
**Introduction**			
Background/rationale	2	Explain the scientific background and rationale for the investigation being reported	3-5
Objectives	3	State specific objectives, including any prespecified hypotheses	5
**Methods**			
Study design	4	Present key elements of study design early in the paper	5
Setting	5	Describe the setting, locations, and relevant dates, including periods of recruitment, exposure, follow-up, and data collection	5-9
Participants	6	(*a*) Give the eligibility criteria, and the sources and methods of selection of participants	5-7
Variables	7	Clearly define all outcomes, exposures, predictors, potential confounders, and effect modifiers. Give diagnostic criteria, if applicable	5-7
Data sources/ measurement	8*	For each variable of interest, give sources of data and details of methods of assessment (measurement). Describe comparability of assessment methods if there is more than one group	*5-7*
Bias	9	Describe any efforts to address potential sources of bias	7-9
Study size	10	Explain how the study size was arrived at	7-9
Quantitative variables	11	Explain how quantitative variables were handled in the analyses. If applicable, describe which groupings were chosen and why	7-9
Statistical methods	12	(*a*) Describe all statistical methods, including those used to control for confounding	7-9
		(*b*) Describe any methods used to examine subgroups and interactions	7-9
		(*c*) Explain how missing data were addressed	7-9
		(*d*) If applicable, describe analytical methods taking account of sampling strategy	
		(*e*) Describe any sensitivity analyses	
**Results**			
Participants	13*	(a) Report numbers of individuals at each stage of study—eg numbers potentially eligible, examined for eligibility, confirmed eligible, included in the study, completing follow-up, and analysed	9
		(b) Give reasons for non-participation at each stage	9
		(c) Consider use of a flow diagram	
Descriptive data	14*	(a) Give characteristics of study participants (eg demographic, clinical, social) and information on exposures and potential confounders	9-13
		(b) Indicate number of participants with missing data for each variable of interest	9
Outcome data	15*	Report numbers of outcome events or summary measures	9-13
Main results	16	(*a*) Give unadjusted estimates and, if applicable, confounder-adjusted estimates and their precision (eg, 95% confidence interval). Make clear which confounders were adjusted for and why they were included	10-13

For peer review only

		(*b*) Report category boundaries when continuous variables were categorized	10-13
		(*c*) If relevant, consider translating estimates of relative risk into absolute risk for a meaningful time period	
Other analyses	17	Report other analyses done—eg analyses of subgroups and interactions, and sensitivity analyses	13-14
**Discussion**			
Key results	18	Summarise key results with reference to study objectives	14
Limitations	19	Discuss limitations of the study, taking into account sources of potential bias or imprecision. Discuss both direction and magnitude of any potential bias	18
Interpretation	20	Give a cautious overall interpretation of results considering objectives, limitations, multiplicity of analyses, results from similar studies, and other relevant evidence	14-17
Generalisability	21	Discuss the generalisability (external validity) of the study results	14-17
**Other information**			
Funding	22	Give the source of funding and the role of the funders for the present study and, if applicable, for the original study on which the present article is based	3

*Give information separately for exposed and unexposed groups.

Note: An Explanation and Elaboration article discusses each checklist item and gives methodological background and published examples of transparent reporting. The STROBE checklist is best used in conjunction with this article (freely available on the Web sites of PLoS Medicine at http://www.plosmedicine.org/, Annals of Internal Medicine at http://www.annals.org/, and Epidemiology at http://www.epidem.com/). Information on the STROBE Initiative is available at www.strobe-statement.org.

For peer review only
